# Author Correction: Efficient electron transfer across hydrogen bond interfaces by proton-coupled and -uncoupled pathways

**DOI:** 10.1038/s41467-019-10193-1

**Published:** 2019-05-02

**Authors:** Tao Cheng, Dong Xue Shen, Miao Meng, Suman Mallick, Lijiu Cao, Nathan J. Patmore, Hong Li Zhang, Shan Feng Zou, Huo Wen Chen, Yi Qin, Yi Yang Wu, Chun Y. Liu

**Affiliations:** 1 Department of Chemistry, Jinan University, 601 Huang-Pu Avenue West, 510632 Guangzhou, China; 2 Department of Chemical Sciences, University of Huddersfield, Queensgate, Huddersfield, HD1 3DH UK

**Keywords:** Chemical bonding, Electron transfer

Correction to: *Nature Communications* 10.1038/s41467-019-09392-7, published online 4 April 2019.

The original version of this Article contained an error in the eighteenth sentence of the third paragraph of the ‘Determination of *H*_ab_ and *k*_ET_ data for the Mo_2_ dimers’ section of the Results, which incorrectly read ‘For **1a**^+^ with 2*H*_ab_ » *k*_B_*T*, it is inappropriate to calculate the rate constant using *H*_ab_ for the PCET reaction in the nonadiabatic regime.’ The correct version states ‘2*H*_ab_ >> *k*_B_*T* ’ in place of ‘2*H*_ab_ » *k*_B_*T*’.

Also, the third sentence of the Discussion originally incorrectly read ‘The linear relationship of ln(*k*_ET_) vs. *r*_ab_ gives *β* = 1.25 (Supplementary Fig. 9).’ The correct version states ‘*R*_ab_’ instead of ‘*r*_ab_’.

This has been corrected in both the PDF and HTML versions of the Article.

