# Author Correction: Efficient electron transfer across hydrogen bond interfaces by proton-coupled and -uncoupled pathways

**DOI:** 10.1038/s41467-019-10580-8

**Published:** 2019-06-04

**Authors:** Tao Cheng, Dong Xue Shen, Miao Meng, Suman Mallick, Lijiu Cao, Nathan J. Patmore, Hong Li Zhang, Shan Feng Zou, Huo Wen Chen, Yi Qin, Yi Yang Wu, Chun Y. Liu

**Affiliations:** 1 Department of Chemistry, Jinan University, 601 Huang-Pu Avenue West, 510632 Guangzhou, China; 2 Department of Chemical Sciences, University of Huddersfield, Queensgate, Huddersfield, HD1 3DH UK

**Keywords:** Chemical bonding, Electron transfer

Correction to: *Nature Communications* 10.1038/s41467-019-09392-7, published online 04 April 2019.

In the first corrected version of this Article, the third sentence of the Discussion incorrectly read ‘The linear relationship of ln(*k*_ET_) vs. *R*_ab_ gives *β* = 1.25’. The corrected version states ‘1.25 Å^−1^’ instead of ‘1.25’. This has now been corrected in both the PDF and HTML versions of the Article.

