# Supplementary material for: Efficient electron transfer across hydrogen bond interfaces by proton-coupled and -uncoupled pathways
Source: Nat Commun. 2019 Apr 4;10:1531. doi: 10.1038/s41467-019-09392-7 (PMC6449364; doi:10.1038/s41467-019-09392-7)
Supplement: Supplementary file 1 — Supplementary Information [file 41467_2019_9392_MOESM1_ESM.docx]

**Efficient electron transfer across hydrogen bond interface by proton-coupled and uncoupled pathways**

Cheng et al.

SUPPLEMENTARY FIGURES

**
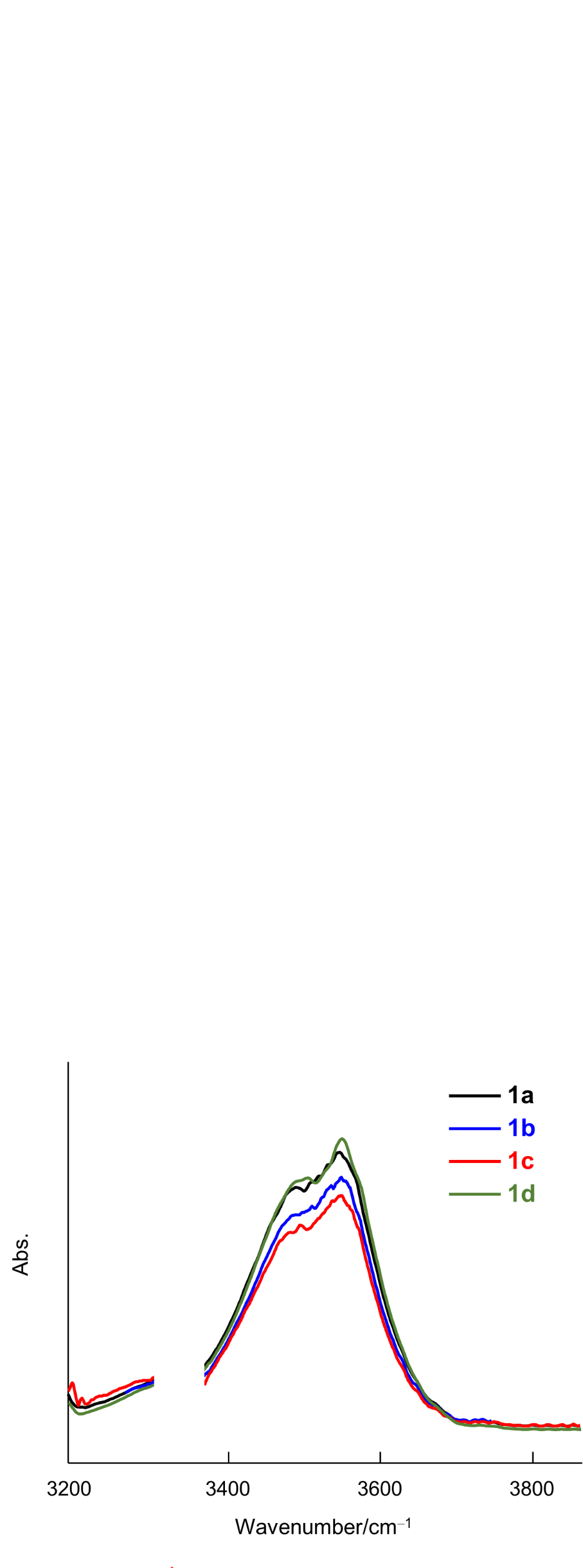
**

**Supplementary Figure 1.** IR spectra of **1a**−**d** in DMF solutions showing the region of the amidate group N−H vibrational band.

**
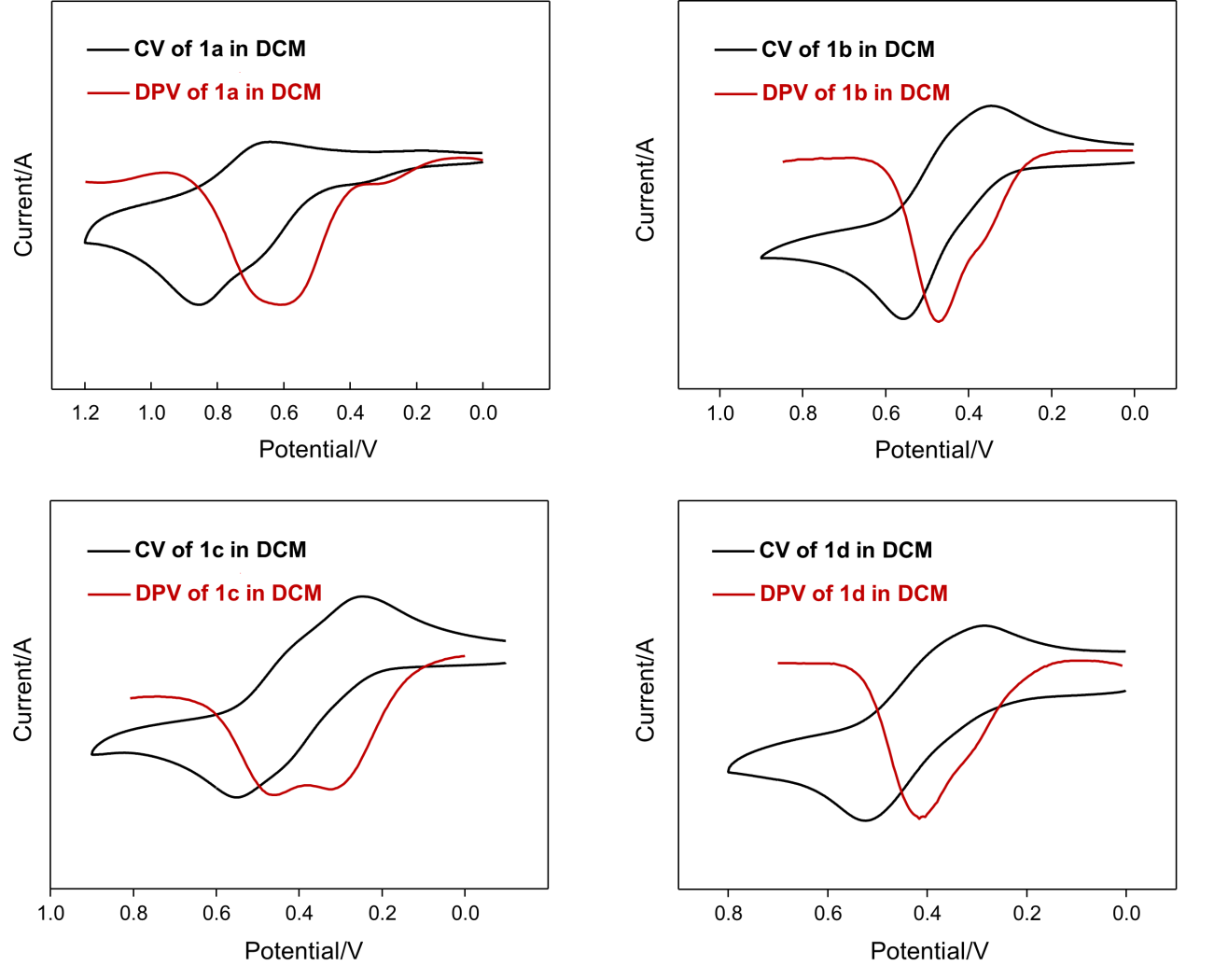
**

**Supplementary Figure 2.** Cyclic voltammograms (CVs, black) and differential pulse voltammograms (DPVs, red) for complexes **1a**−**d** in 0.1M *^n^*Bu_4_NPF_6_ **/** DCM solutions. Under these electrochemical conditions, the potential for ferrocene, *E*_1/2_(Fc^+/0^), is 0.52 V. For **1a**, the higher potential wave (~0.80 V) is due to the redox processes of the N(CH_3_)_2_ groups, which makes the redox processes of the Mo_2_ centers irreversible.


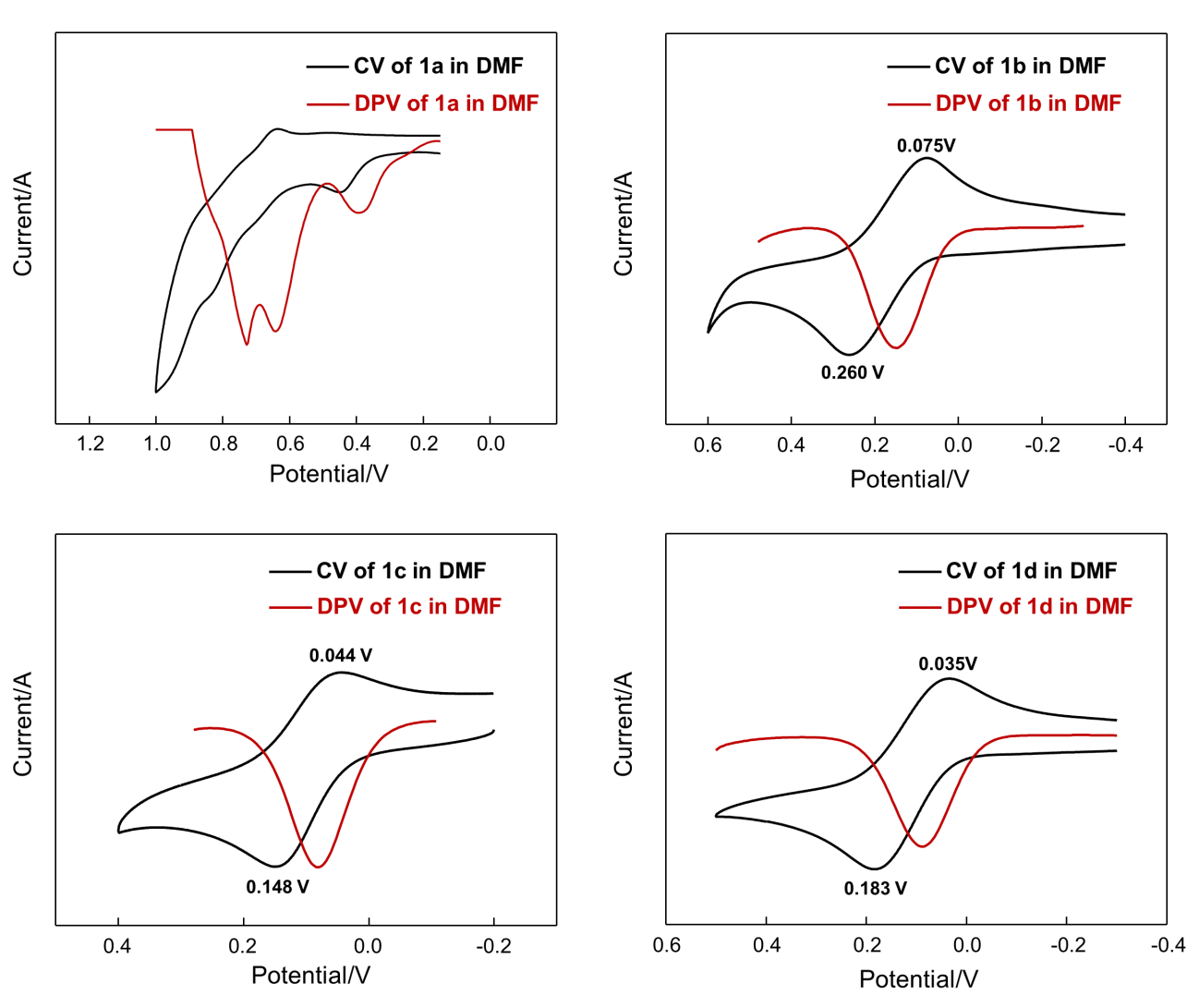


**Supplementary Figure 3.** Cyclic voltammograms (CVs, black) and differential pulse voltammograms (DPVs, red) for **1a**−**d** in 0.1M *^n^*Bu_4_NPF_6_ **/** DMF solutions, showing only one redox couple for the corresponding Mo_2_ monomers. Under these electrochemical conditions, the potential for ferrocene, *E*_1/2_(Fc^+/0^), is 0.52 V. For **1a**, the higher potential waves (0.6 − 0.8 V) are due to the redox processes of the N(CH_3_)_2_ groups, which makes the redox processes (~ 0.4 V) of the Mo_2_ center irreversible.

**
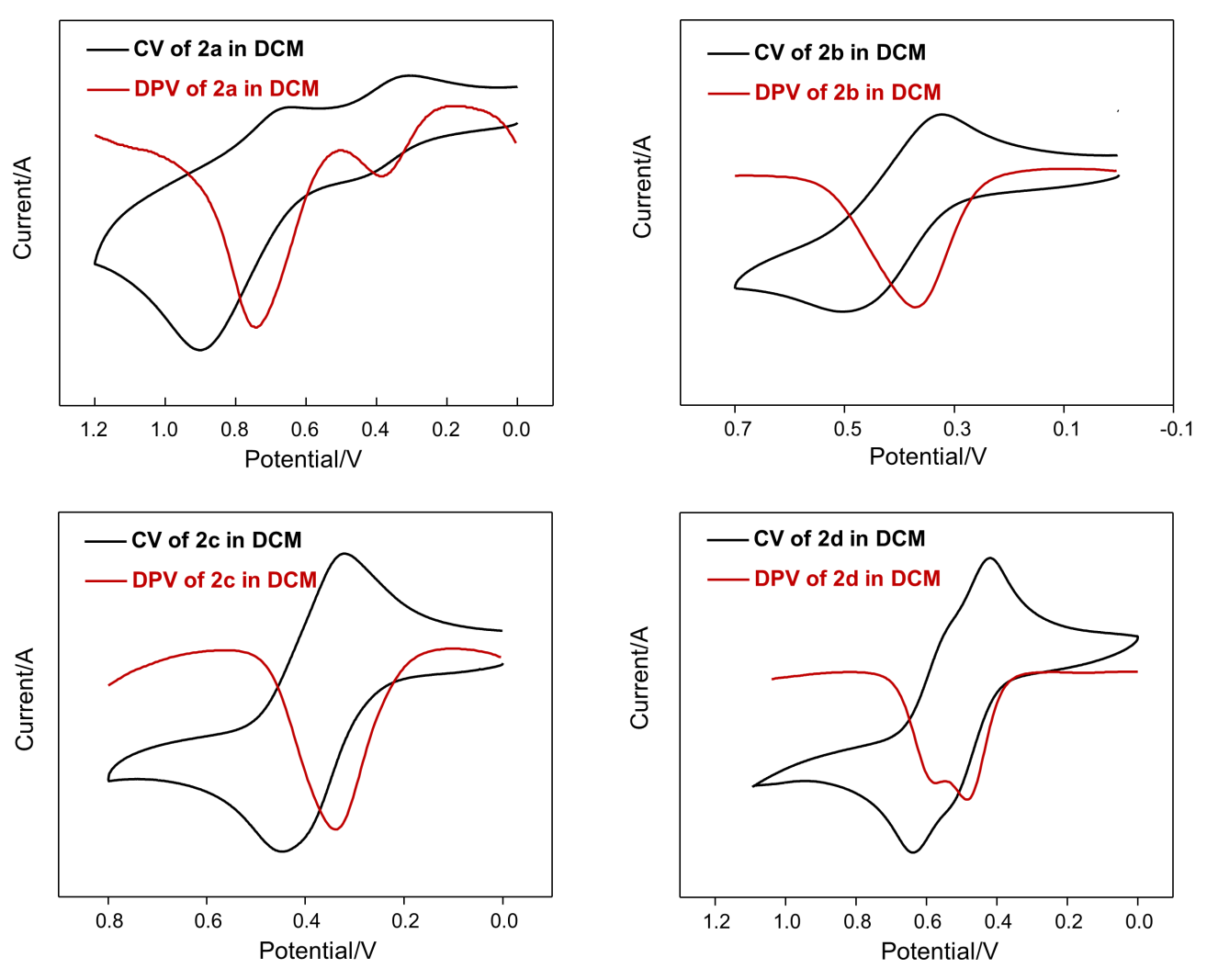
**

**Supplementary Figure 4.** Cyclic voltammograms (CVs, black) and differential pulse voltammograms (DPVs, red) for complexes **2a**−**d** in 0.1M *^n^*Bu_4_NPF_6_ **/** DCM solutions. Under these electrochemical conditions, the potential for ferrocene, *E*_1/2_(Fc^+/0^), is 0.52 V. For **2a**, the higher potential wave (~0.80 V) is due to the redox processes of the N(CH_3_)_2_ groups and the lower potential wave (~0.40 V) corresponds to the redox processes occurring on the Mo_2_ centers.


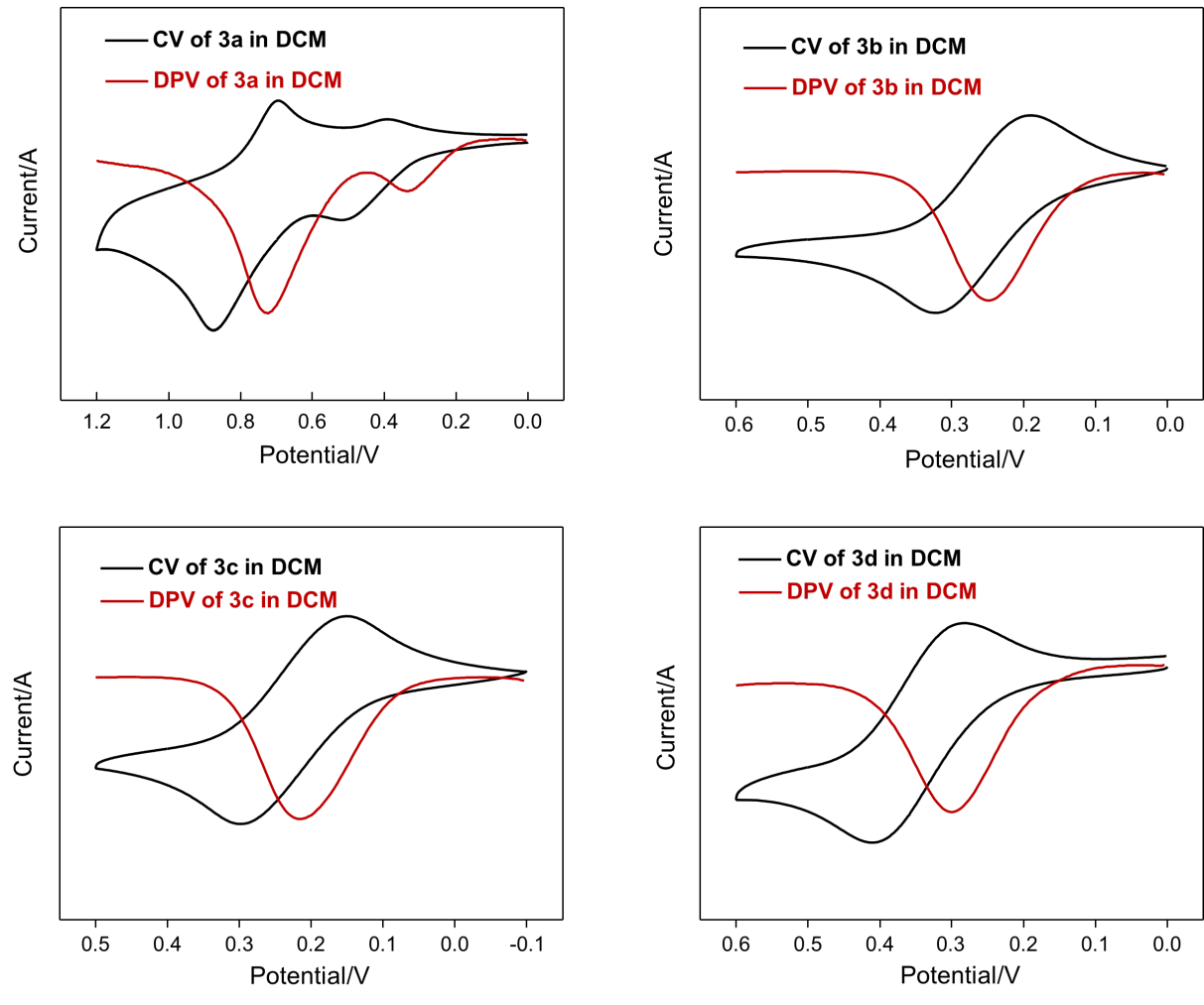


**Supplementary Figure 5.** Cyclic voltammograms (CVs, black) and differential pulse voltammograms (DPVs, red) for complexes **3a**−**d** in 0.1M *^n^*Bu_4_NPF_6_ **/** DCM solutions. Under these electrochemical conditions, the potential for ferrocene, *E*_1/2_(Fc^+/0^), is 0.52 V. Complex **3a** exhibits two redox waves. The higher potential wave (~0.80 V) is due to the redox processes of the N(CH_3_)_2_ groups and the lower potential wave (~0.40 V) corresponds to the redox process occurring on the Mo_2_ centers.


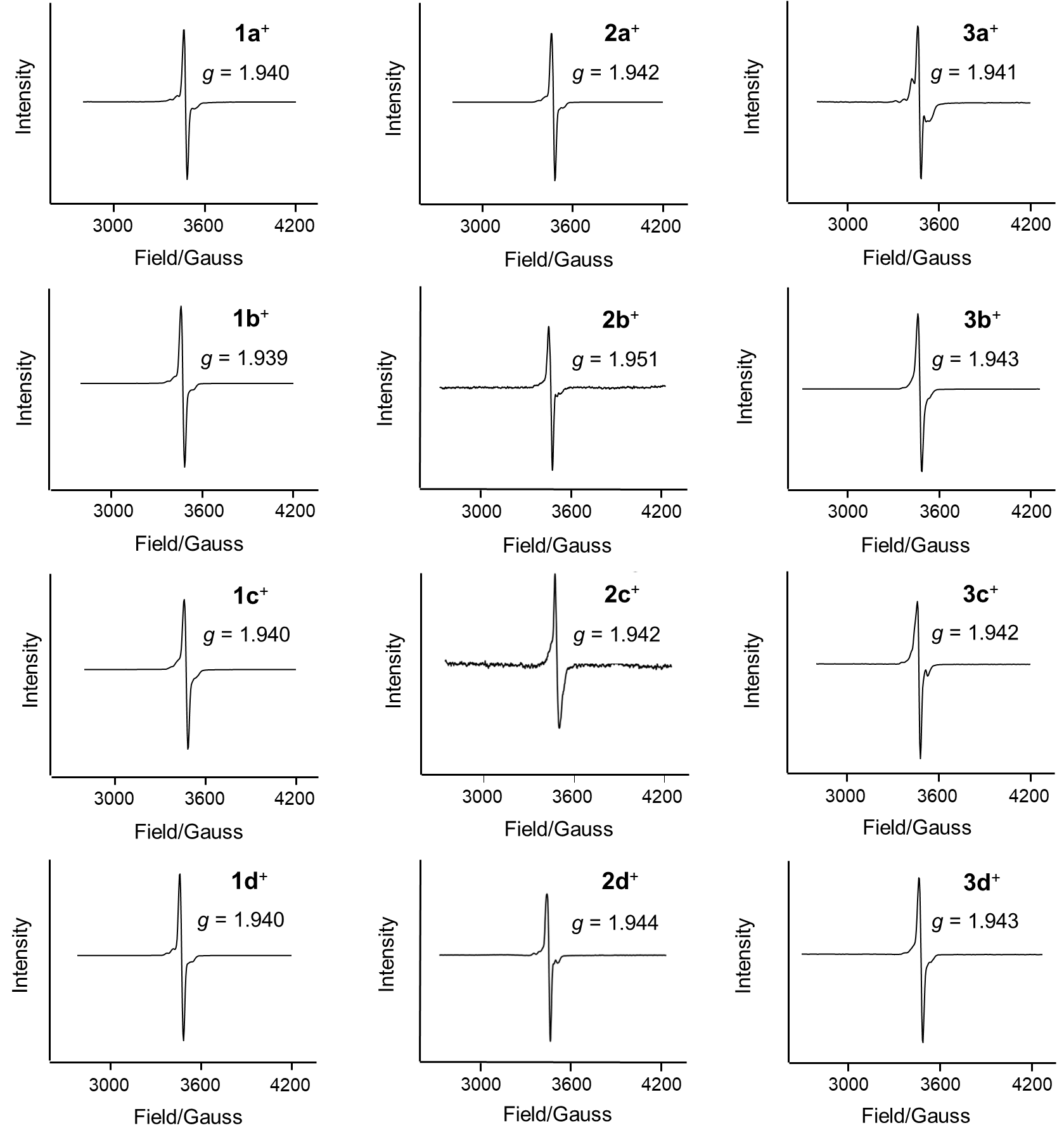


**Supplementary Figure 6.** EPR spectra for mixed-valence complexes (**1a**−**d)**^+^, (**2a**−**d)**^+^ and (**3a**−**d)**^+^ in DCM.


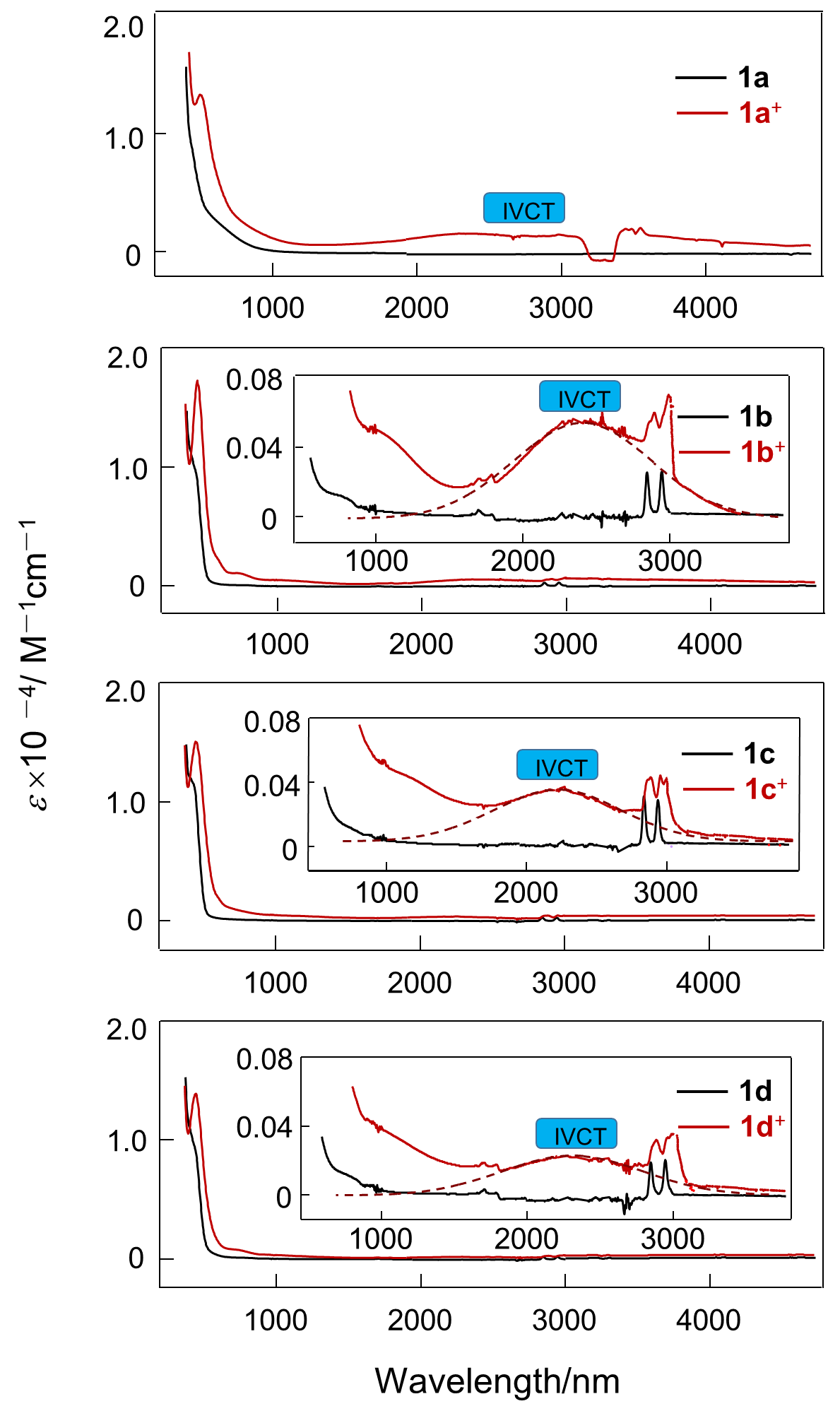


**Supplementary Figure 7.** UV-vis-near-mid-IR spectra for the mixed-valence complexes (red) (**1a**−**d**)^+^ in DCM solutions, in comparison with that for the corresponding neutral precursor **1a**−**d** (black). The insets are expansions of the NIR regions showing the Gaussian-shaped intervalence charge transfer (IVCT) absorption bands.


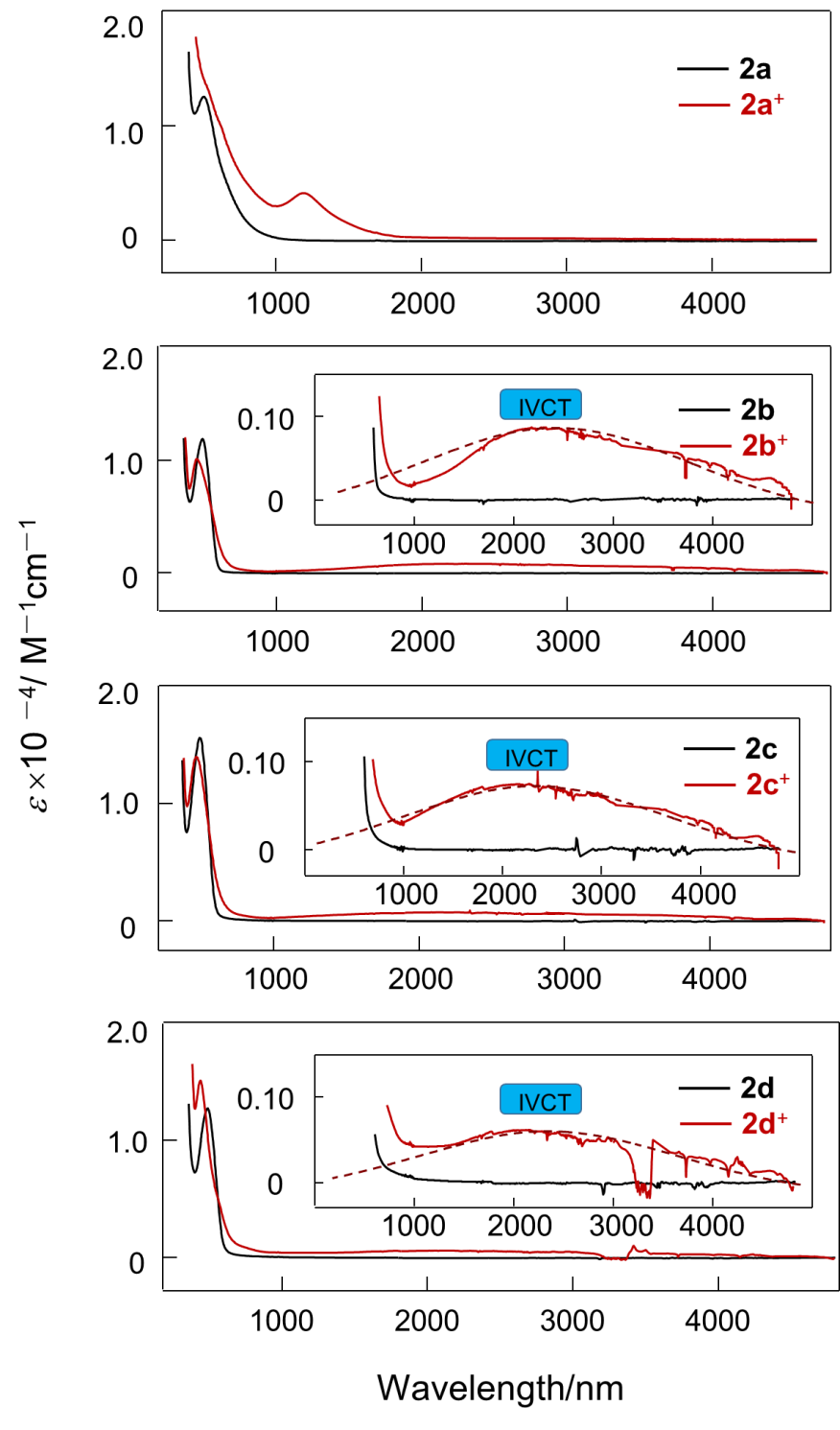


**Supplementary Figure 8.** Vis-near-mid-IR spectra of the mixed-valence complexes (red) for (**2a**−**d**)^+^ in DCM solutions, in comparison with that for the corresponding neutral precursor **2a**−**d** (black). The insets are expansions of the NIR regions showing the Gaussian-shaped intervalence charge transfer (IVCT) absorption bands.

**
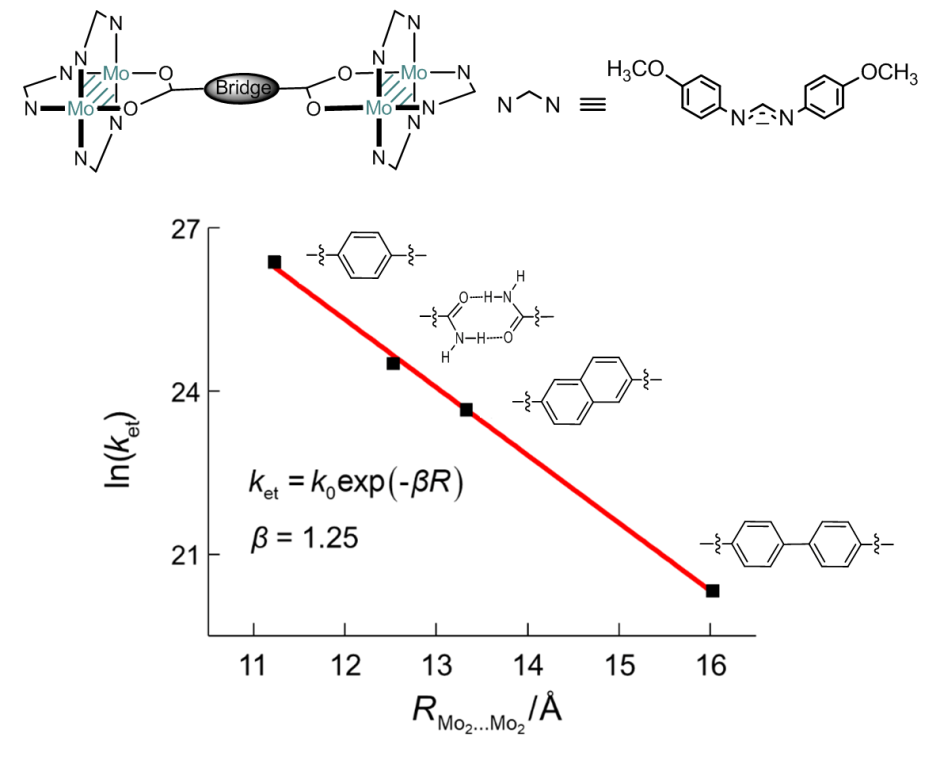
**

**Supplementary Figure 9.** A plot of ln(*k*_et_) versus *R*_Mo2⋅⋅⋅Mo2_ (Å) for four MV Mo_2_ dimers with different bridges, showing the exponential relationship between electron transfer rates (*k*_et_) and distances (*R*). The data fitting to the linear equation are for a series of complexes {[Mo_2_]−bridge−[Mo_2_]}^+^, where [Mo_2_] = [Mo_2_(DAniF)_3_O_2_C] (DAniF = *N*, *N*′-(*p*-dianisyl)formamidinate) and bridge = phenylene, amide-amide hydrogen bonds (this work), naphthalene[[1]](#endnote-1) and biphenylene[[2]](#endnote-2).

**
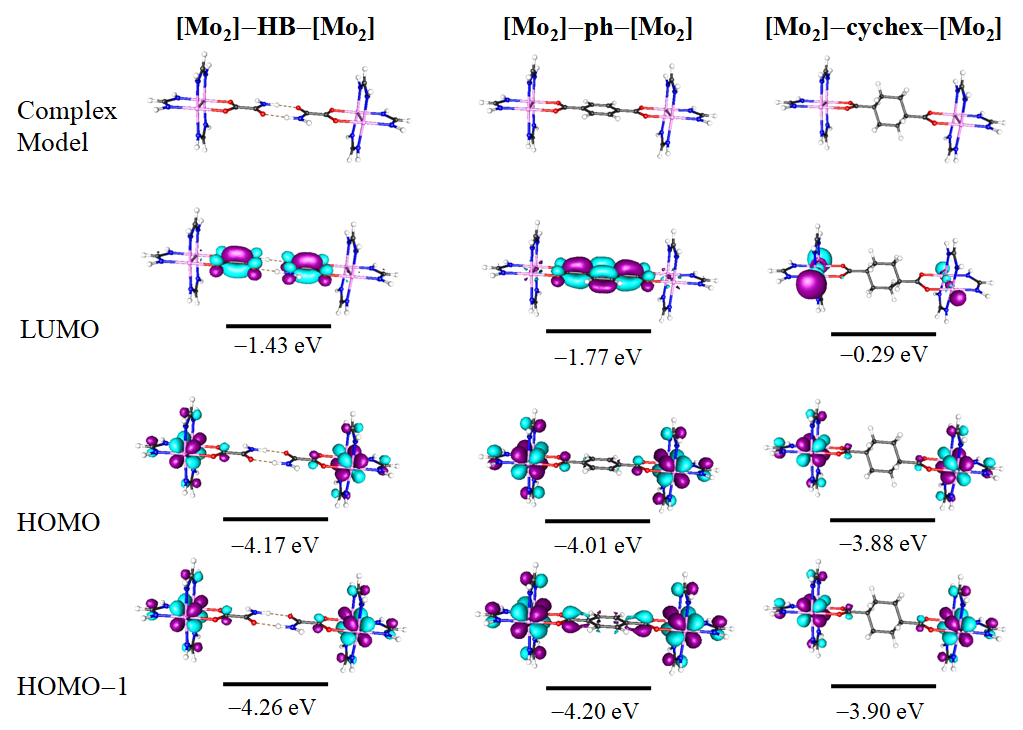
**

**Supplementary Figure 10.** Frontier molecular orbitals, and their corresponding orbital energies, resulting from DFT calculations employing a COSMO dichloromethane solvation models. The computational models are simplified by replacing the aryl groups on the ancillary formamidinate ligands with H atoms, i.e., [(HC{NH}_2_)_3_Mo_2_]−bridge−[Mo_2_({NH}_2_CH)_3_], where bridge = amide-amide hydrogen bonds (**1**), phenylene (**2**) and cyclohexylene (**3**).


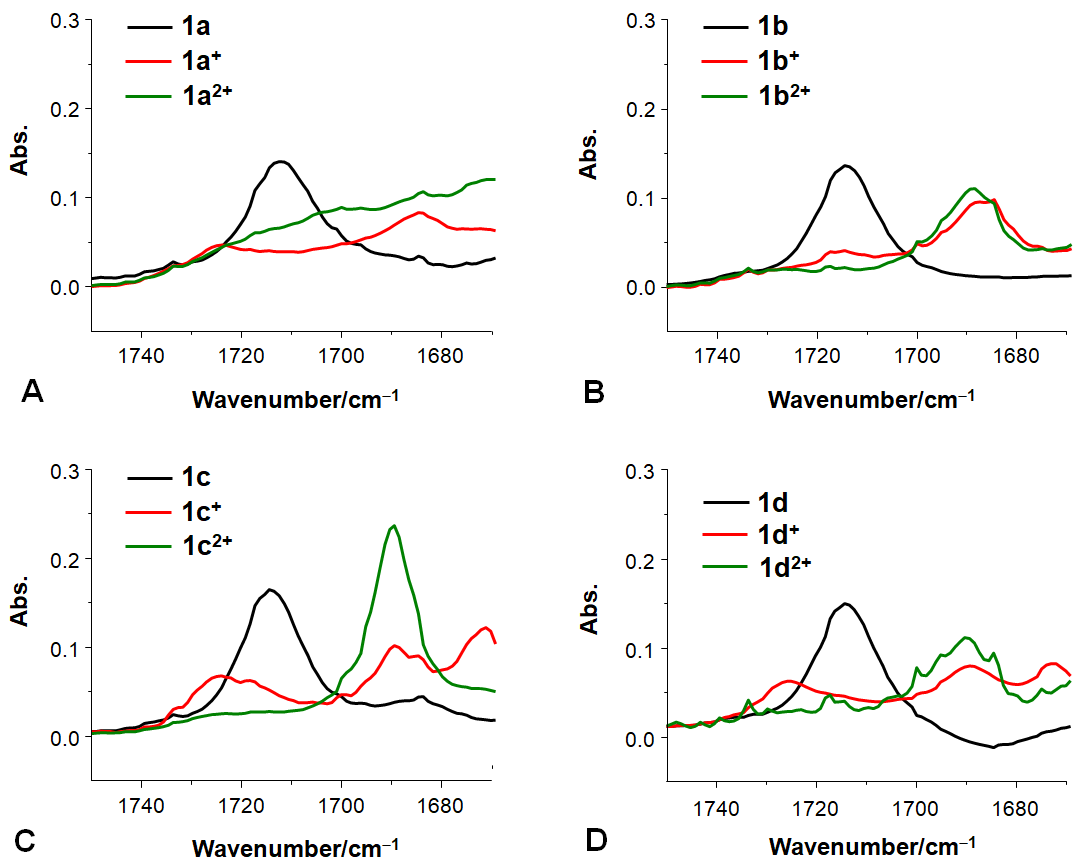


**Supplementary Figure 11.** IR spectra and tentative assignments of the vibrational bands for **1** and **1^2+^** with (**A**) X = N(CH_3_)_2_ (**1a**); (**B**) X = CH(CH_3_)_2_ (**1b**); (**C**) X = OCH_3_ (**1c**) and (**D**) X = CH_3_(**1d**). All the neutral complexes exhibit a C=O stretch at 1735 cm^−1^ for the amide group. Upon oxidation, this band is red shifted to 1687 cm^−1^ typically for the most weakly hydrogen bonded **1c**^2+^ (A); on the other extreme, for the most strongly coupled and hydrogen bonded system **1a**^2+^, the spectrum does not present a C=O stretch character, which is attributed to conversion of C=O to C−OH through proton transfer (C). For the two intermediated species, the vibrational features are attenuated from **1b**^2+^(B) and **1d**^2+^ (D) as the hydrogen bond is weakened (See the text). For all the mixed-valence dimers (**1a**^+^, **1b**^+^, **1c**+ and **1d**^+^) a weak band is observed. Importantly, this band increases in intensity for the dications **1b**^2+^ and **1c**^2+^ but the band feature disappears for **1a**^2+^ and **1d**^2+^. These results are solid evidences supporting proton-coupled (PCET) and proton-uncoupled (PUET) electron transfer pathways.

SUPPLEMENTARY METHODS

**Supplementary Figure 12.** Synthetic route to complexes **1a**−**d**. Starting materials formamidines (ArNCHNHAr)[[3]](#endnote-3) and the dimolybdenum precursors Mo_2_(ArNCHNAr)_3_(O_2_CCH_3_)[[4]](#endnote-4) were synthesized according to published methods.

**1a**: Yield = 0.117g, 52 . ^1^H NMR *δ*(ppm in CDCl_3_): 8.465 (s, 2H, −NC*H*N−), 7.169 (s, 2H, N−*H*), 6.492 (d, 16H, aromatic C−*H*), 6.471(d, 16H, aromatic C−*H*), 6.345 (d, 8H, aromatic C−*H*), 6.274 (d, 8H, aromatic C−H), 5.588(d, 2H, N−*H*), 2.822 (s, 48H, −C*H*_3_), 2.776 (s, 24H, −C*H*_3_). ^1^H NMR *δ*(ppm in DMSO-*d*_6_): 8.475 (s, 2H, −NC*H*N−), 8.461 (s, 1H, −NC*H*N−), 8.040 (s, 1H, N−*H*), 7.833(s, 1H, N−*H*), 6.717 (d, 4H, aromatic C−*H*), 6.482 (S, 16H, aromatic C−*H*), 6.236 (s, 4H, aromatic C−*H*), 2.771 (s, 24H, −C*H*_3_), 2.702 (s, 12H, −C*H*_3_).


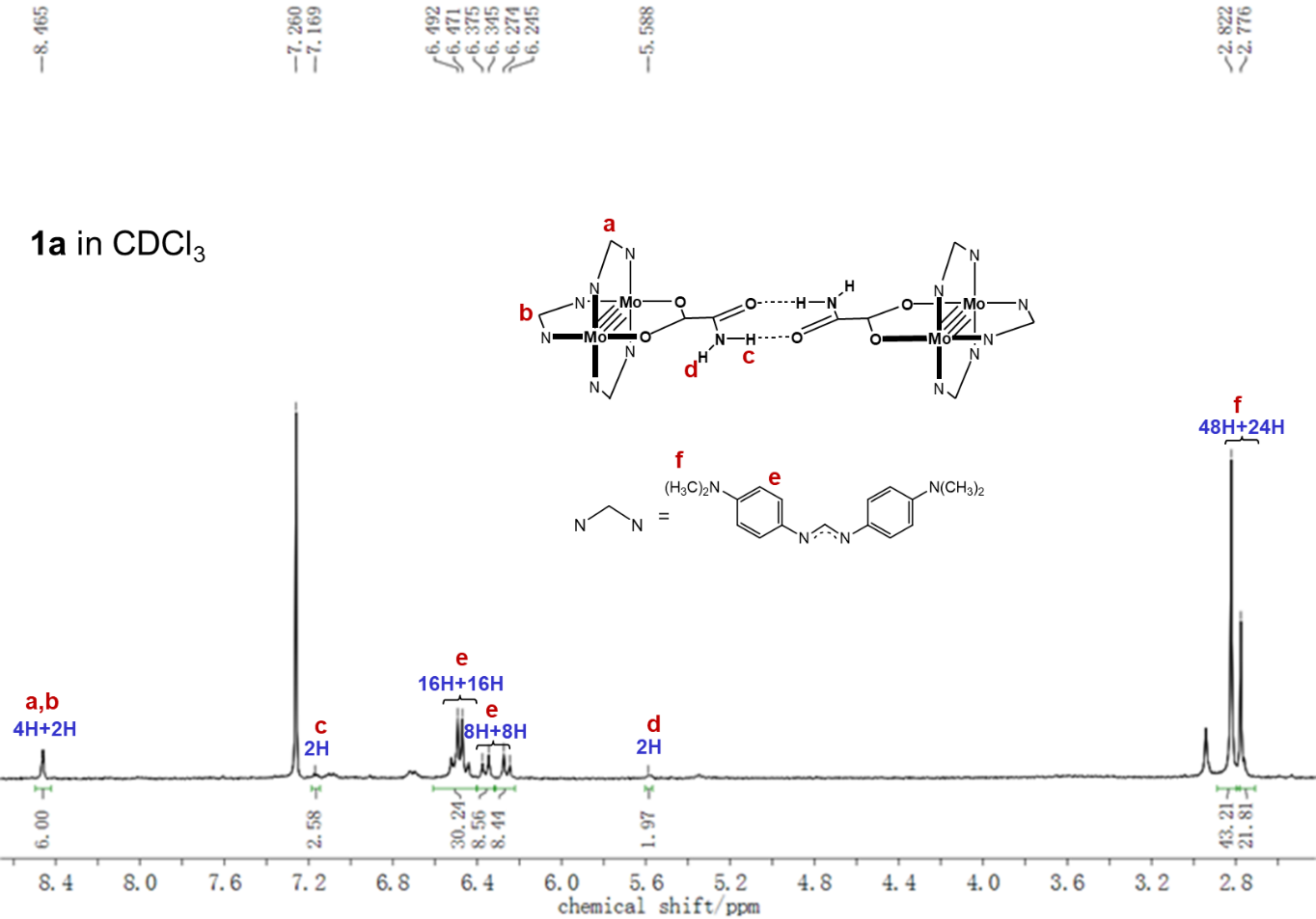


**Supplementary Figure 13.** ^1^H NMR spectrum of **1a** in CDCl_3_.


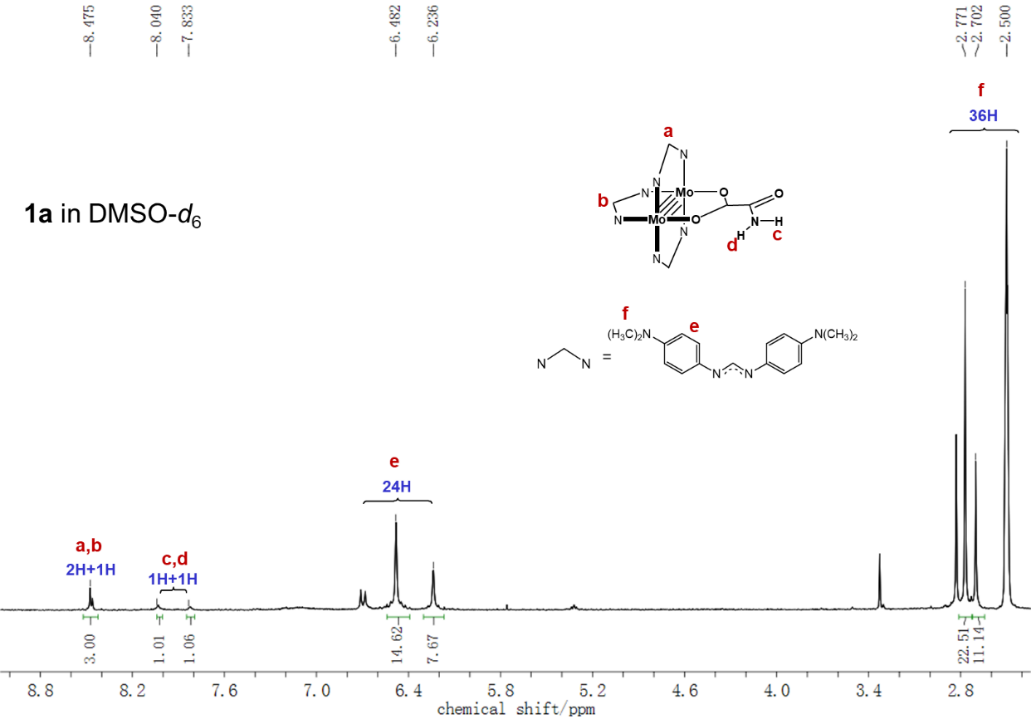


**Supplementary Figure 14.** ^1^H NMR spectrum of **1a** in DMSO-*d_6_*.

**1b**: Yield = 0.119g, 53 . ^1^H NMR *δ*(ppm in CDCl_3_): 8.570 (s, 4H, −NC*H*N−), 8.523 (s, 2H, −NC*H*N−), 7.196 (d, 2H, N−*H*), 6.899 (d, 16H, aromatic C−*H*), 6.700 (d, 8H, aromatic C−H), 6.438 (d, 16H, aromatic C−*H*), 6.151 (d, 8H, aromatic C−*H*), 5.647 (d, 2H, N−*H*), 2.788 (m, 12H, −C*H*(CH_3_)_2_), 1.186 (d, 48H, −C*H*_3_), 1.126 (s, 24H, −CH(C*H*_3_)_2_). ^1^H NMR *δ*(ppm in DMSO-*d*_6_): 8.598 (s, 2H, −NC*H*N−), 8.593 (s, 1H, −NC*H*N−), 8.149 (s, 1H, N−*H*), 7.876 (s, 1H, N−*H*), 6.915 (d, 8H, aromatic C−*H*), 6.662 (s, 4H, aromatic C−*H*), 6.503 (s, 8H, aromatic C−*H*), 6.206 (d, 4H, aromatic C−*H*), 2.765 (m, 6H, −C*H*(CH_3_)_2_), 3.356 (d, 12H, −C*H*_3_), 1.128 (d, 36H, −CH(C*H*_3_)_2_).


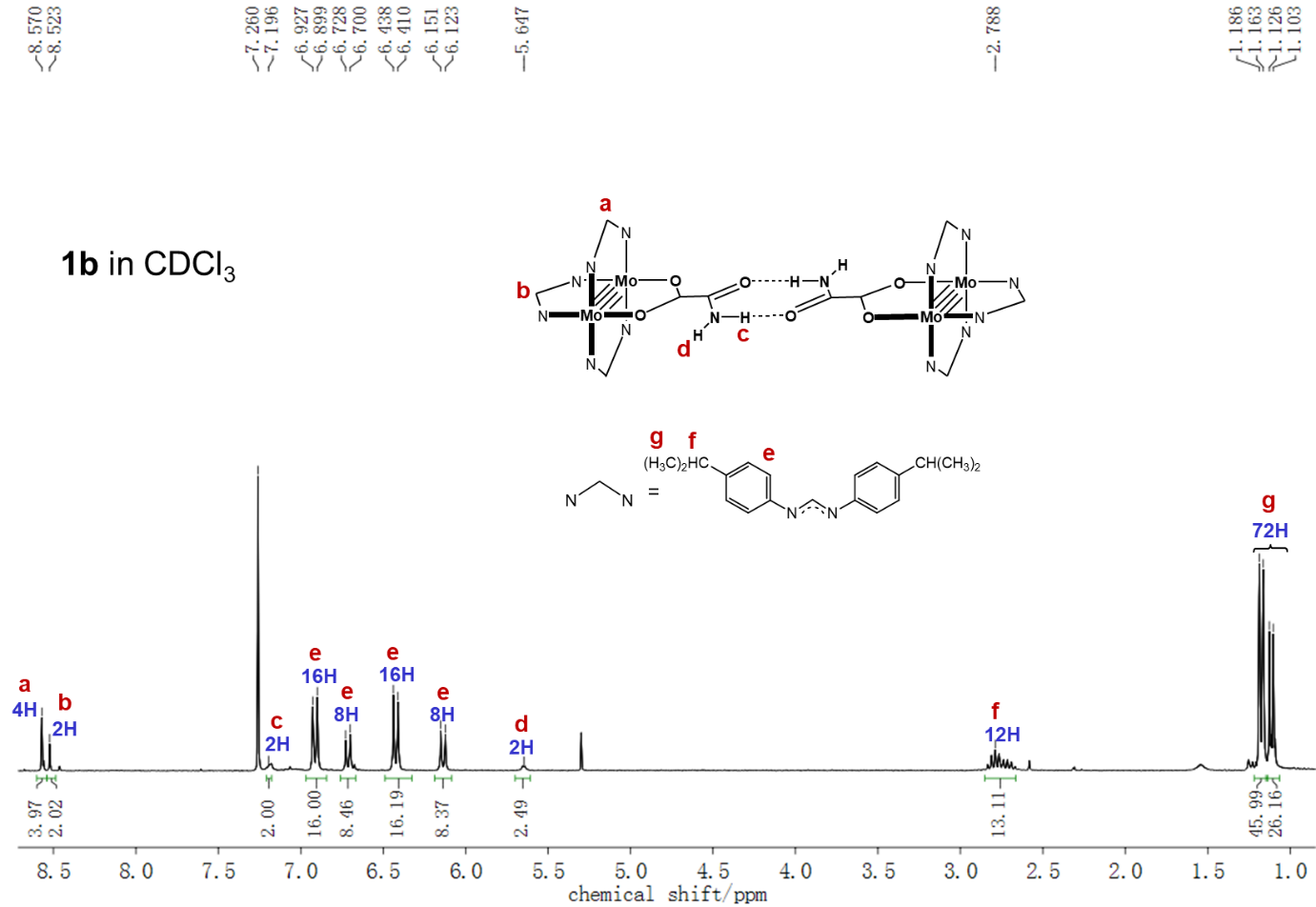


**Supplementary Figure 15.** ^1^H NMR spectrum of **1b** in CDCl_3_.


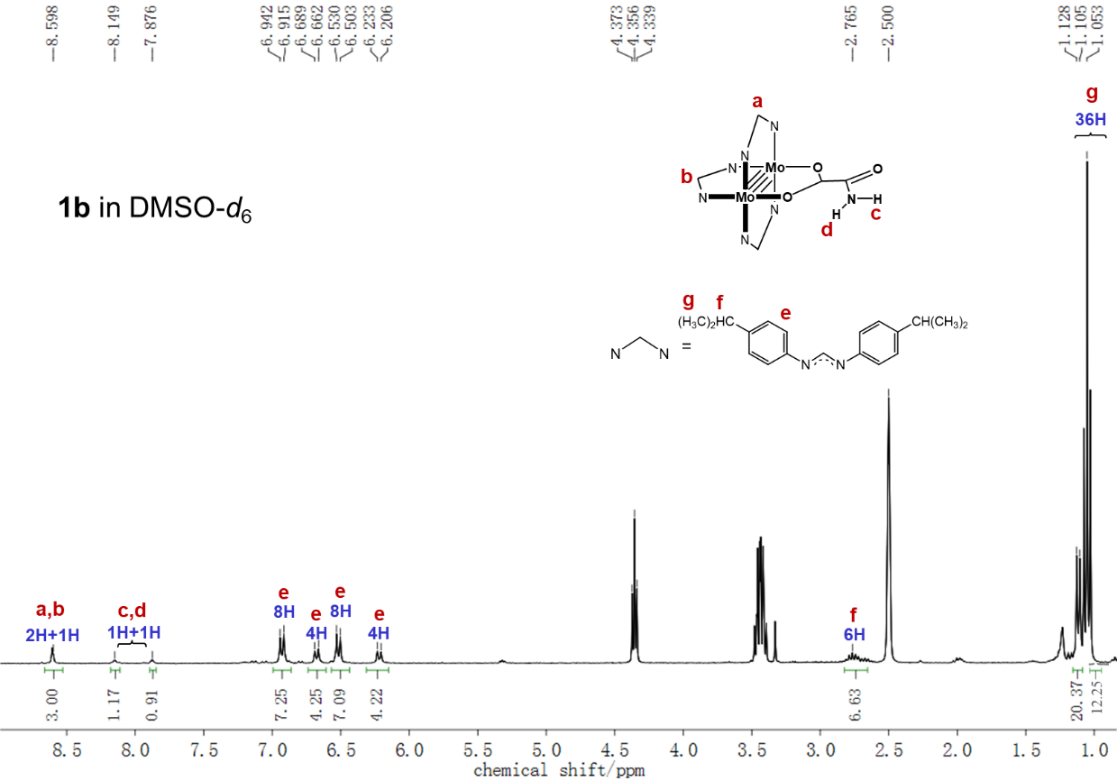


**Supplementary Figure 16.** ^1^H NMR spectrum of **1b** in DMSO-*d*_6_.

**1c**: Yield = 0.128g, 61 . ^1^H NMR *δ*(ppm in CDCl_3_): 8.464 (s, 4H, −NC*H*N−), 8.445 (s, 2H, −NC*H*N−), 7.169 (d, 2H, N−*H*), 6.621 (d, 16H, aromatic C−*H*), 6.522 (d, 16H, aromatic C−*H*), 6.433 (d, 8H, aromatic C−*H*), 6.229 (d, 8H, aromatic C−*H*), 5.703(d, 2H, N−*H*), 3.719 (s, 24H, −C*H*_3_), 3.661 (s, 12H, −C*H*_3_). ^1^H NMR *δ*(ppm in DMSO-*d*_6_): 8.499 (s, 1H, −NC*H*N−), 8.464 (s, 2H, −NC*H*N−), 8.095 (s, 1H, N−*H*), 7.874 (s, 1H, N−*H*), 6.650 (d, 8H, aromatic C−*H*), 6.616 (s, 8H, aromatic C−*H*), 6.408 (s, 4H, aromatic C−*H*), 6.330 (d, 4H, aromatic C−*H*), 3.654 (s, 12H, −C*H*_3_), 3.586 (s, 6H, −C*H*_3_).


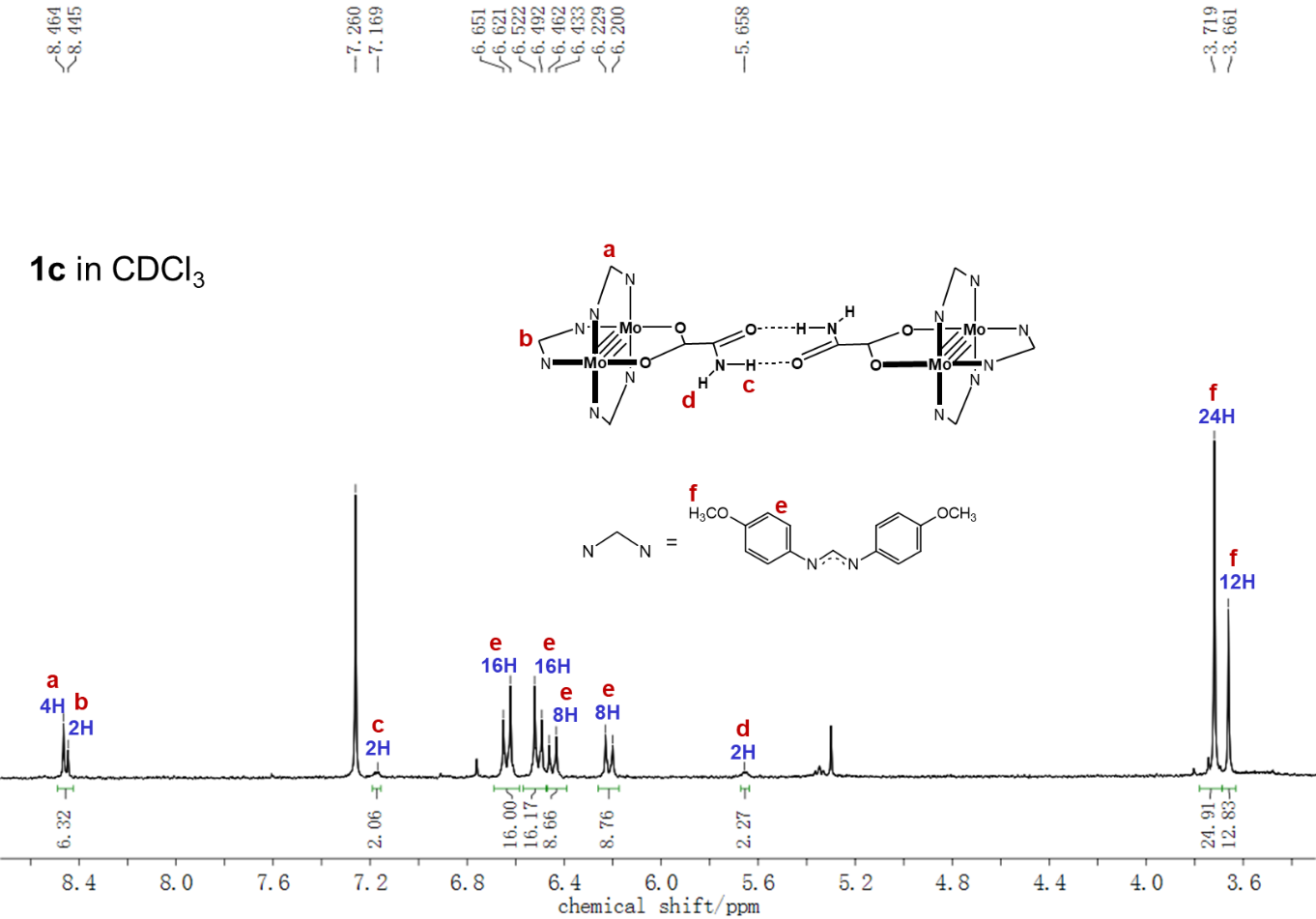


**Supplementary Figure 17.** ^1^H NMR spectrum of **1c** in CDCl_3_.


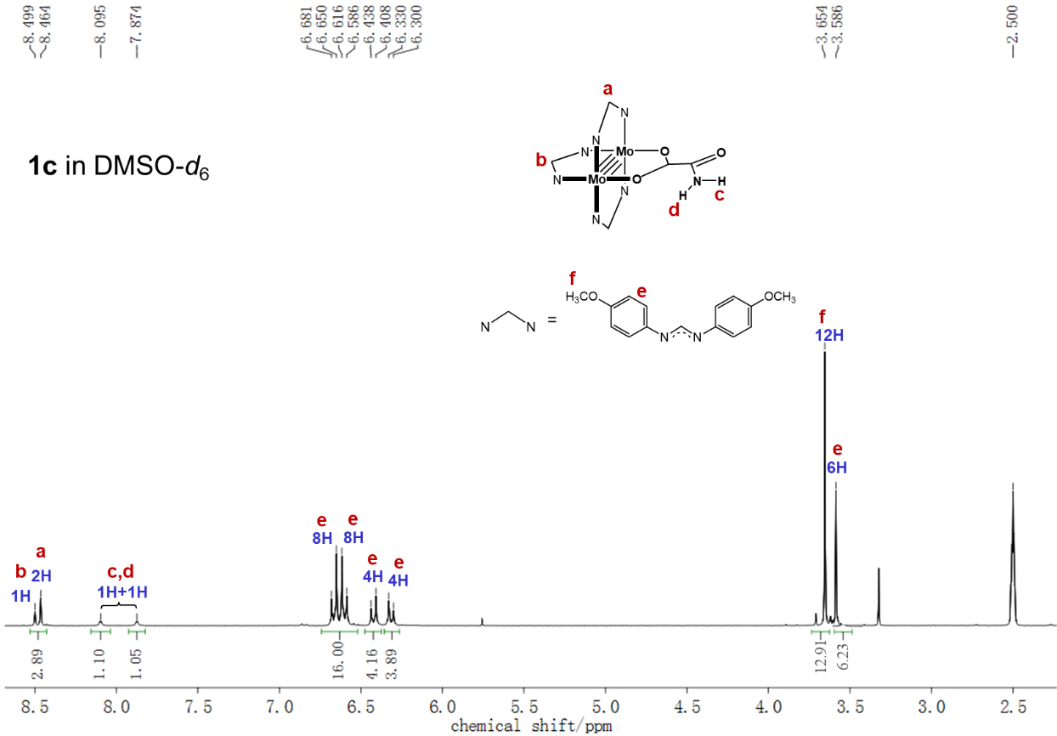


**Supplementary Figure 18.** ^1^H NMR spectrum of **1c** in DMSO-*d*_6_.

**1d**: Yield = 0.125g, 66 . ^1^H NMR *δ*(ppm in CDCl_3_): 8.557 (s, 4H, −NC*H*N−), 8.508 (s, 2H, −NC*H*N−), 7.175 (d, 2H, N−*H*), 6.856 (d, 16H, aromatic C−*H*), 6.669 (d, 8H, aromatic C−H), 6.451 (d, 16H, aromatic C−*H*), 6.175 (d, 8H, aromatic C−*H*), 5.652(d, 2H, N−*H*), 2.226 (s, 24H, −C*H*_3_), 2.160 (s, 12H, −C*H*_3_). ^1^H NMR *δ*(ppm in DMSO-*d*_6_): 8.598 (s, 2H, −NC*H*N−), 8.575 (s, 1H, −NC*H*N−), 8.142 (s, 1H, N−*H*), 7.864 (s, 1H, N−*H*), 6.877 (d, 8H, aromatic C−*H*), 6.632 (S, 4H, aromatic C−*H*), 6.545 (s, 8H, aromatic C−*H*), 6.262 (d, 4H, aromatic C−*H*), 2.183 (s, 12H, −C*H*_3_), 2.093 (s, 6H, −C*H*_3_).


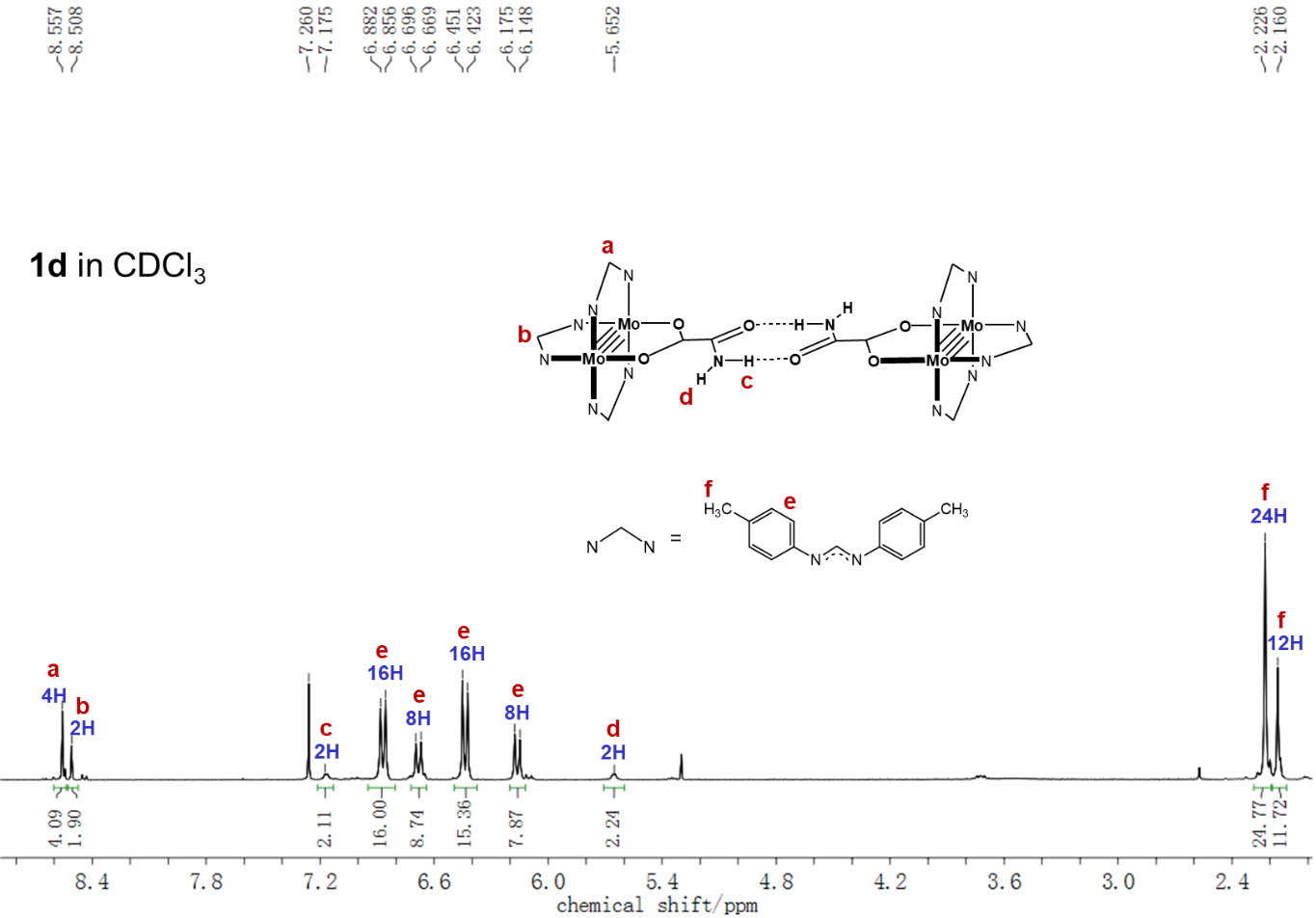


**Supplementary Figure 19.** ^1^H NMR spectrum of **1d** in CDCl_3_.


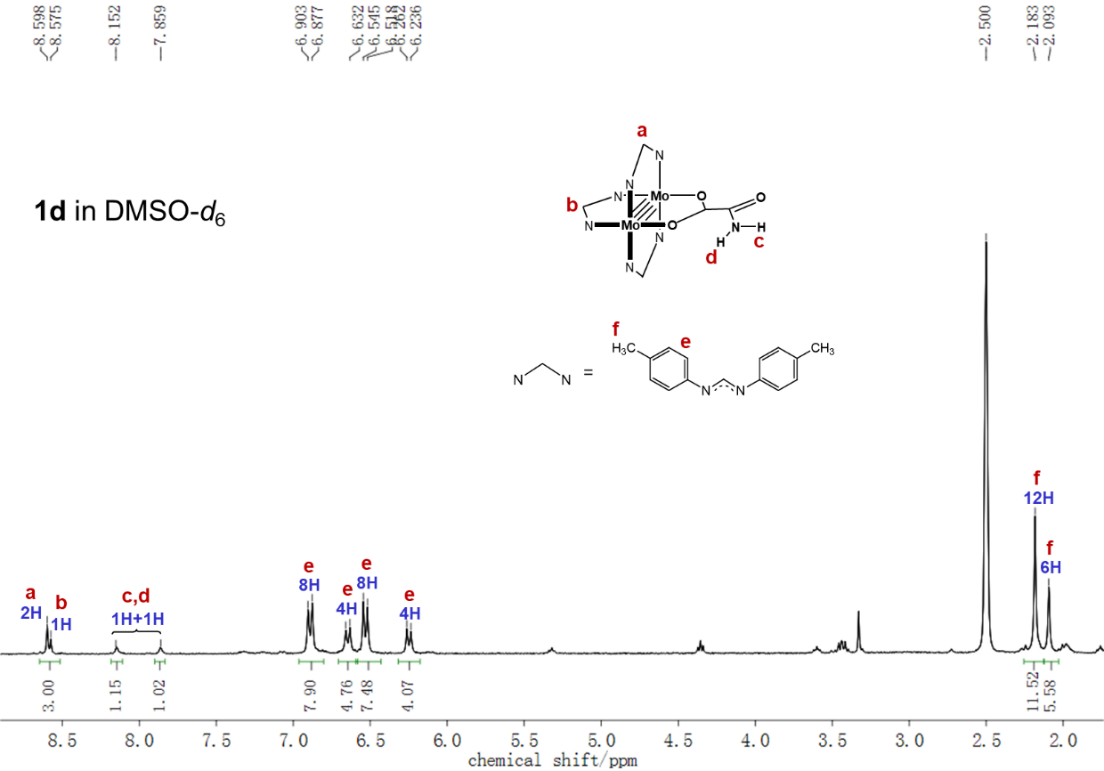


**Supplementary Figure 20.** ^1^H NMR spectrum of **1d** in DMSO-*d*_6_.

**Supplementary Figure 21.** Synthetic route for complexes **2a**−**d**.

**2a**: Yield = 0.145g, 65 . ^1^H NMR *δ*(ppm in CDCl_3_): 8.524 (s, 2H, −NC*H*N−), 8.346 (s, 4H, −NC*H*N−), 8.282 (s, 4H, aromatic C−*H*), 6.524 (s, 32H, aromatic C−*H*), 6.377 (d, 8H, aromatic C−*H*), 6.345 (d, 8H, aromatic C−*H*), 2.817 (s, 48H, −C*H*_3_), 2.781 (s, 24H, −C*H*_3_).


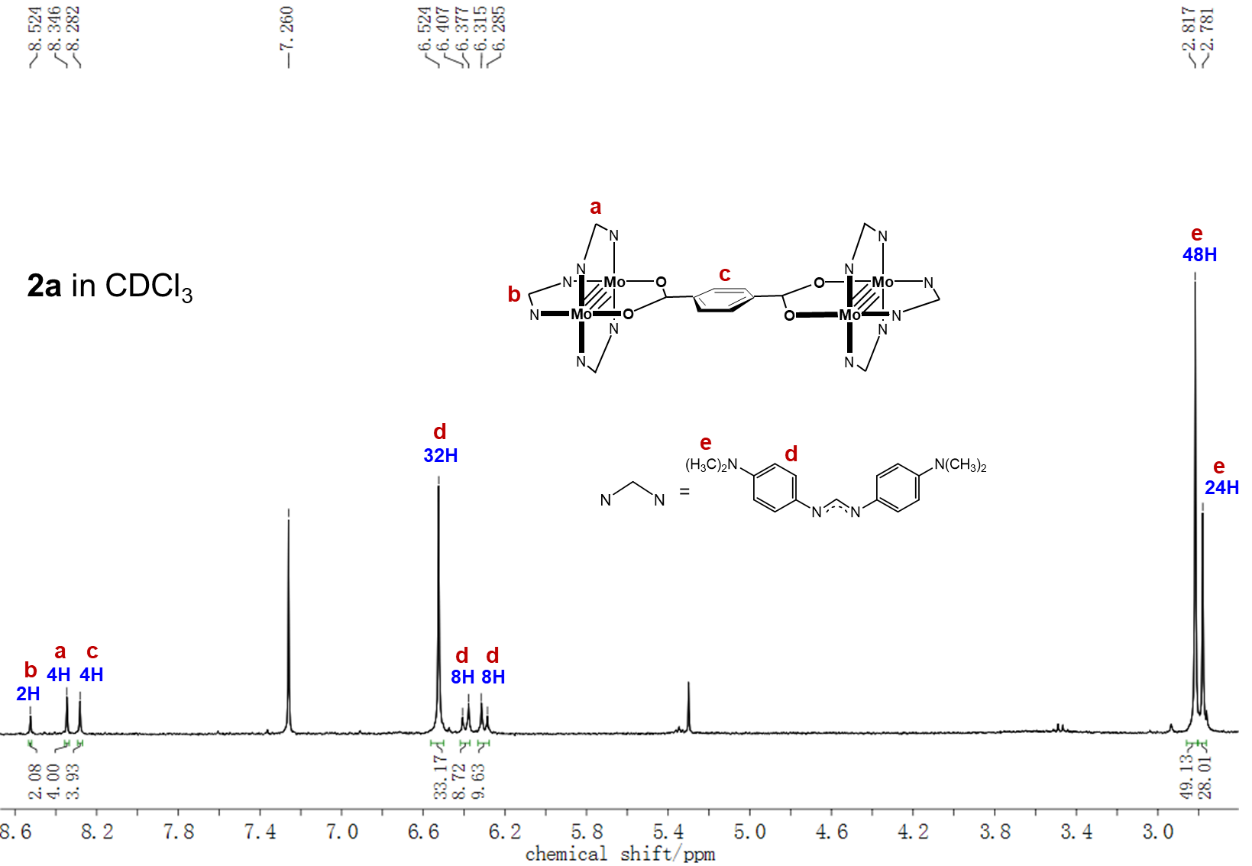


**Supplementary Figure 22.** ^1^H NMR spectrum of complex **2a** in CDCl_3_.

**2b**: Yield = 0.156g, 70 . ^1^H NMR *δ*(ppm in CDCl_3_): 8.562 (s, 2H, −NC*H*N−), 8.465 (s, 4H, −NC*H*N−), 8.330 (s, 4H, aromatic C−*H*), 6.911 (d, 16H, aromatic C−*H*), 6.701 (d, 8H, aromatic C−*H*), 6.506 (d, 16H, aromatic C−*H*), 6.178 (d, 8H, aromatic C−*H*), 2.787 (m, 8H, −C*H*(CH_3_)_2_), 2.728 (m, 4H, −C*H*(CH_3_)_2_), 1.182 (d, 48H, −C*H*_3_), 1.112 (d, 24H, −C*H*_3_).


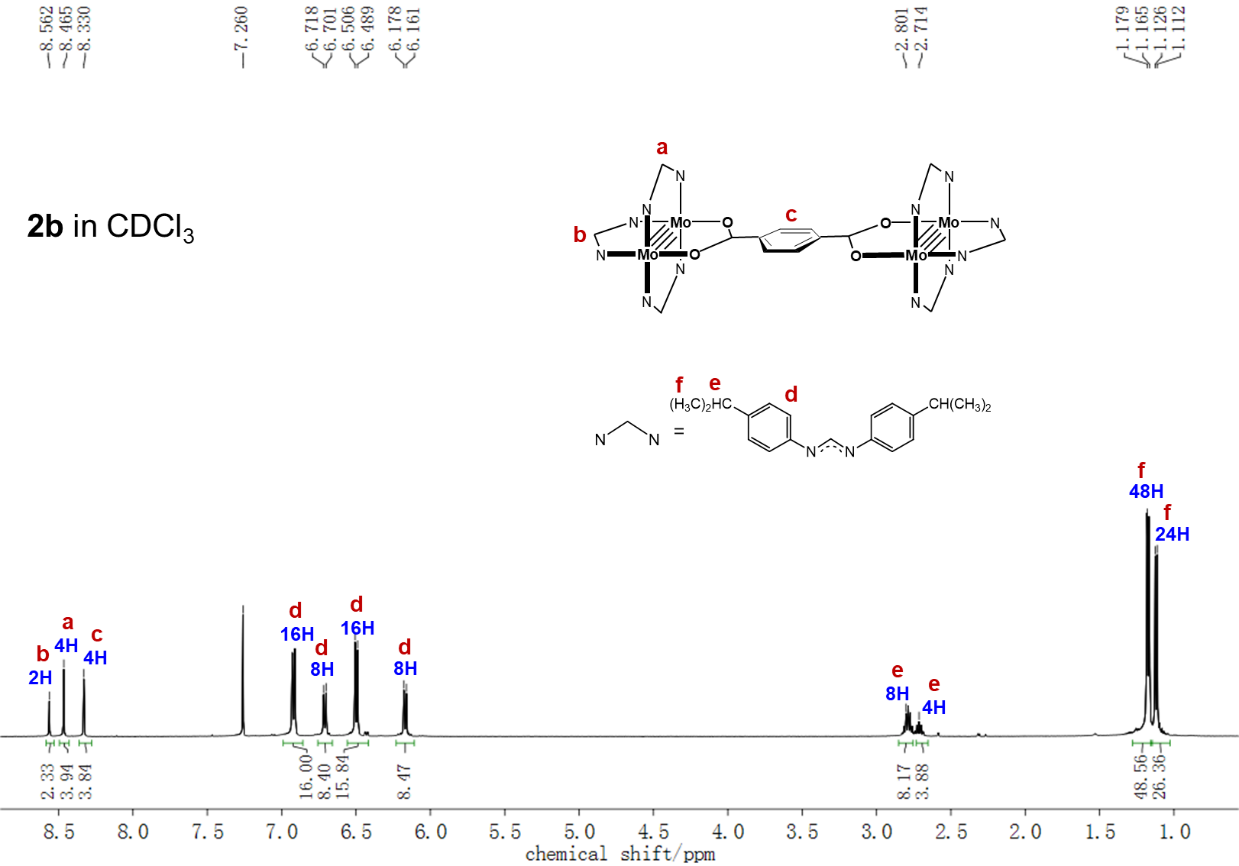


**Supplementary Figure 23.** ^1^H NMR spectrum of complex **2b** in CDCl_3_.

**2c**: Yield = 0.150g, 72 . ^1^H NMR *δ*(ppm in CDCl_3_): 8.493 (s, 2H, −NC*H*N−), 8.357 (s, 4H, −NC*H*N−), 8.352 (s, 4H, aromatic C−*H*), 6.636 (d, 16H, aromatic C−*H*), 6.576 (d, 16H, aromatic C−*H*), 6.455 (d, 8H, aromatic C−*H*), 6.266 (d, 8H, aromatic C−*H*), 3.719 (s, 24H, −C*H*_3_), 3.672 (s, 12H, −C*H*_3_)

.
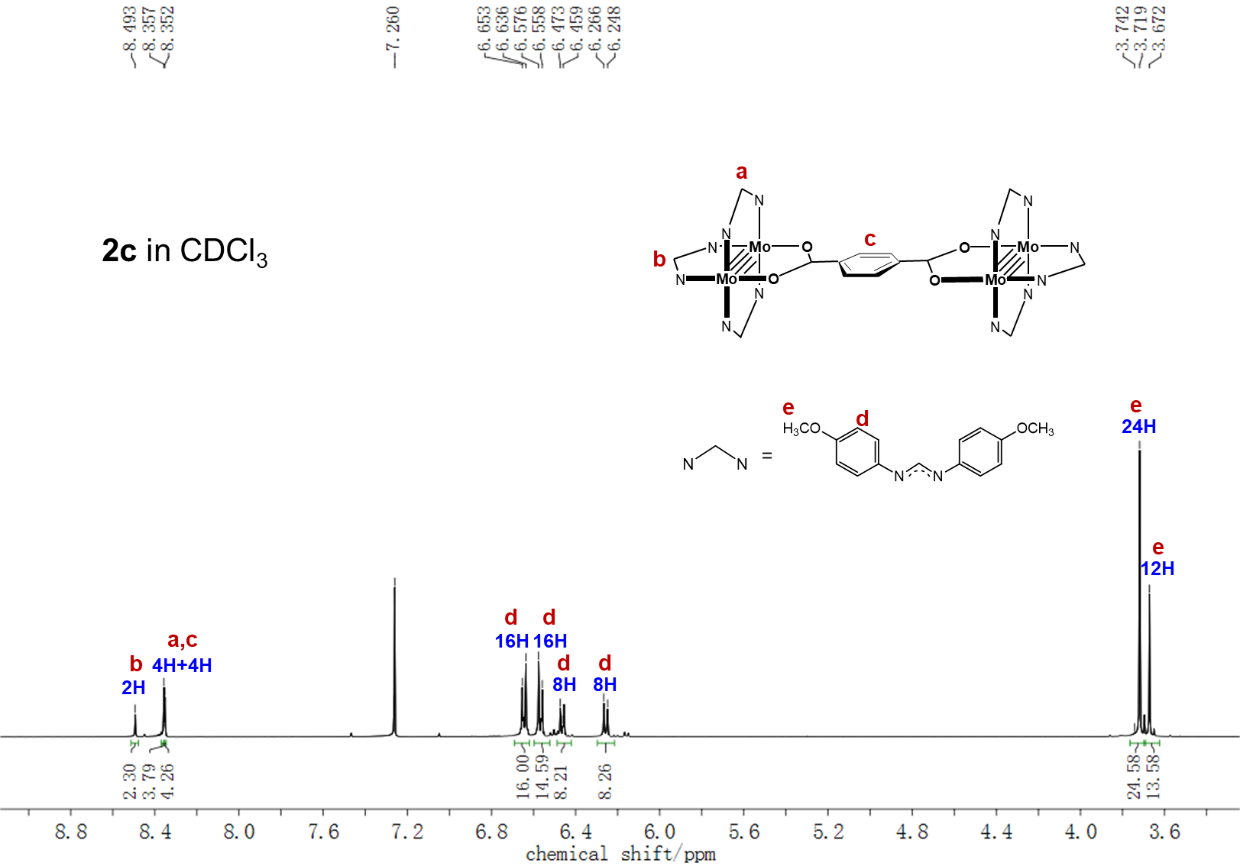


**Supplementary Figure 24.** ^1^H NMR spectrum of complex **2c** in CDCl_3_.

**2d**: Yield = 0.142g, 75 . ^1^H NMR *δ*(ppm in CDCl_3_): 8.562 (s, 2H, −NC*H*N−), 8.443 (s, 4H, −NC*H*N−), 8.312 (s, 4H, aromatic C−*H*), 6.864 (d, 16H, aromatic C−*H*), 6.688 (d, 8H, aromatic C−*H*), 6.499 (d, 16H, aromatic C−*H*), 6.219 (d, 8H, aromatic C−*H*), 2.227 (s, 24H, −C*H*_3_), 2.172 (s, 12H, −C*H*_3_).


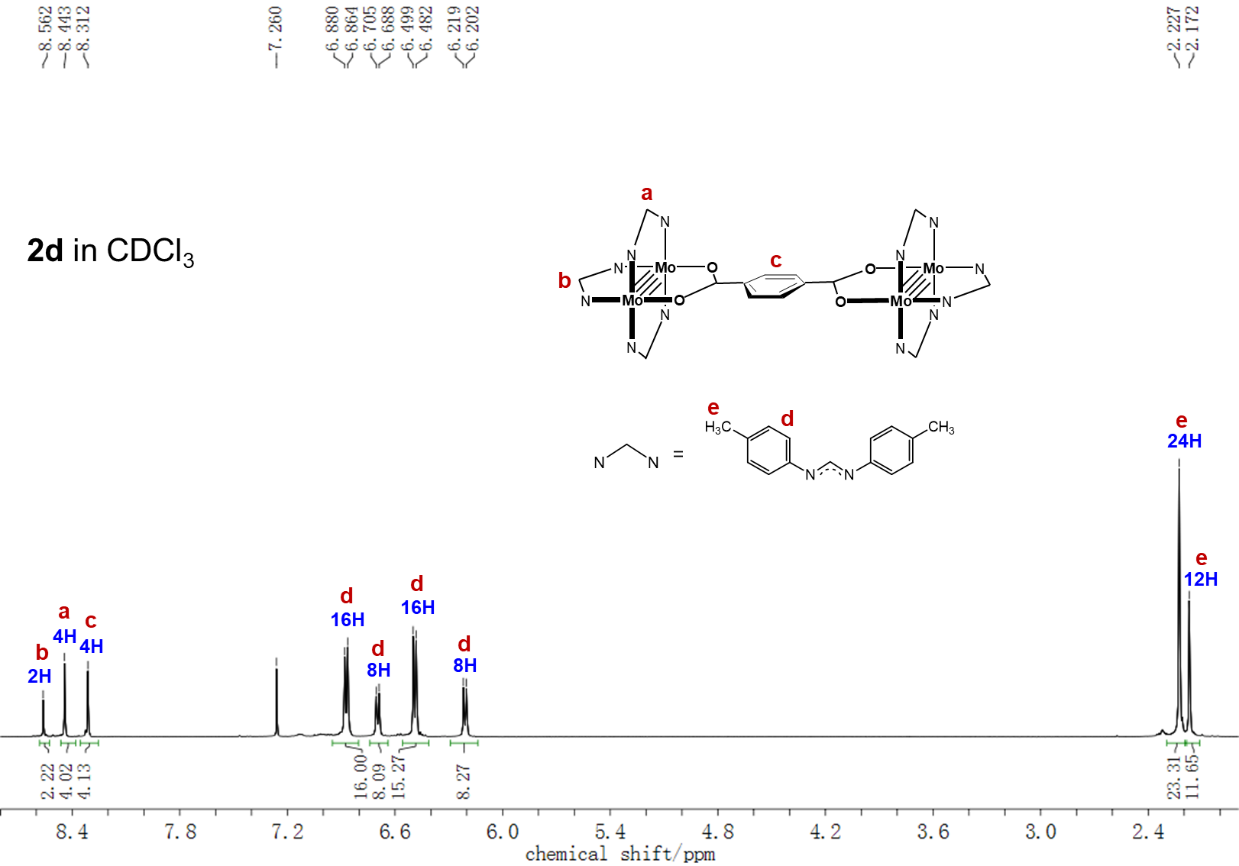


**Supplementary Figure 25.** ^1^H NMR spectrum of complex **2d** in CDCl_3_.

**Supplementary Figure 26.** Synthetic route to complexes **3a**−**d**.

**3a**: Yield = 0.199g, 89 . ^1^H NMR *δ*(ppm in CDCl_3_): 8.463 (s, 2H, −NC*H*N−), 8.366 (s, 4H, −NC*H*N−), 6.515 (d, 16H, aromatic C−*H*), 6.510 (d, 16H, aromatic C−*H*), 6.358 (d, 8H, aromatic C−*H*), 6.282 (d, 8H, aromatic C−*H*), 2.820 (s, 48H, −C*H*_3_), 2.768 (s, 24H, −C*H*_3_), 2.300 (m, 4H, −C*H*_2_), 2.027 (m, 2H, −C*H*), 1.754 (m, 4H, −C*H*_2_).


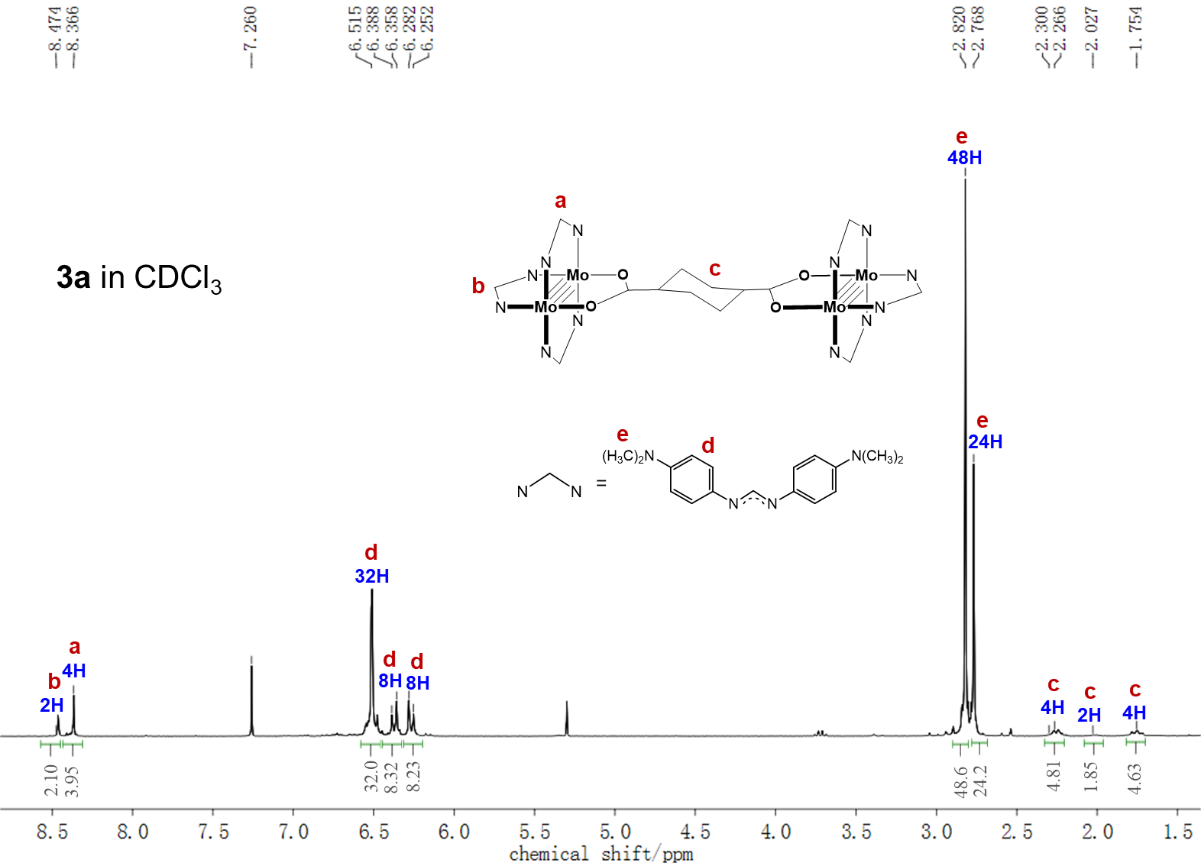


**Supplementary Figure 27.** ^1^H NMR spectrum of complex **3a** in CDCl_3_.

**3b**: Yield = 0.174g, 78 . ^1^H NMR *δ*(ppm in CDCl_3_): 8.500 (s, 2H, −NC*H*N−), 8.470 (s, 4H, −NC*H*N−), 6.698 (d, 16H, aromatic C−*H*), 6.675 (d, 16H, aromatic C−*H*), 6.466 (d, 8H, aromatic C−*H*), 6.141 (d, 8H, aromatic C−*H*), 2.783 (m, 12H, −C*H*(CH_3_)_2_), 1.185 (d, 48H, −C*H*_3_), 1.119 (d, 24H, −C*H*_3_), 2.259 (m, 4H, −C*H*_2_), 2.000 (m, 2H, −C*H*), 1.755 (m, 4H, −C*H*_2_).


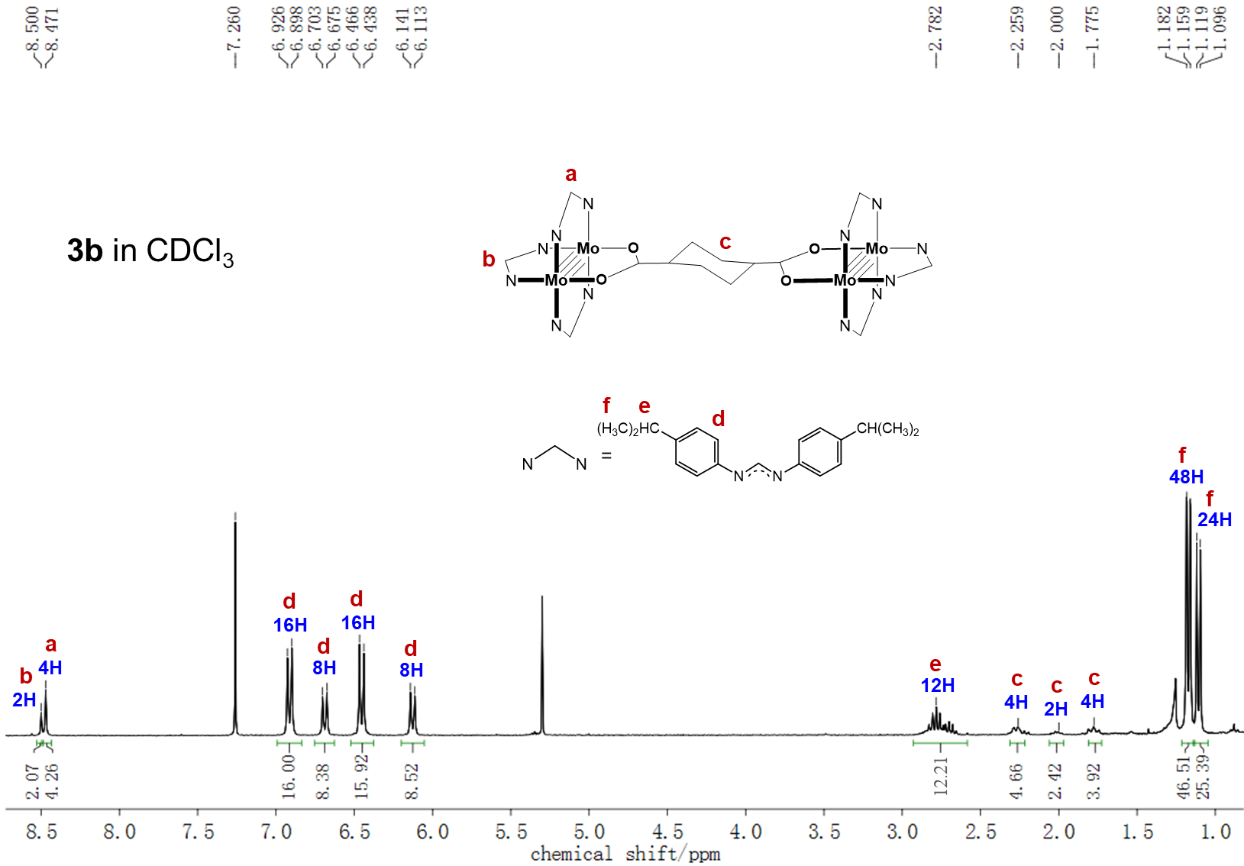


**Supplementary Figure 28.** ^1^H NMR spectrum of complex **3b** in CDCl_3_.

**3c**: Yield = 0.156g, 75 . ^1^H NMR *δ*(ppm in CDCl_3_): 8.435 (s, 2H, −NC*H*N−), 8.376 (s, 4H, −NC*H*N−), 6.629 (d, 16H, aromatic C−*H*), 6.549 (d, 16H, aromatic C−*H*), 6.431 (d, 8H, aromatic C−*H*), 6.241 (d, 8H, aromatic C−*H*), 3.720 (s, 24H, −C*H*_3_), 3.660 (s, 12H, −C*H*_3_), 2.309 (m, 4H, −C*H*_2_), 1.788 (m, 2H, −C*H*), 1.269 (m, 4H, −C*H*_2_).


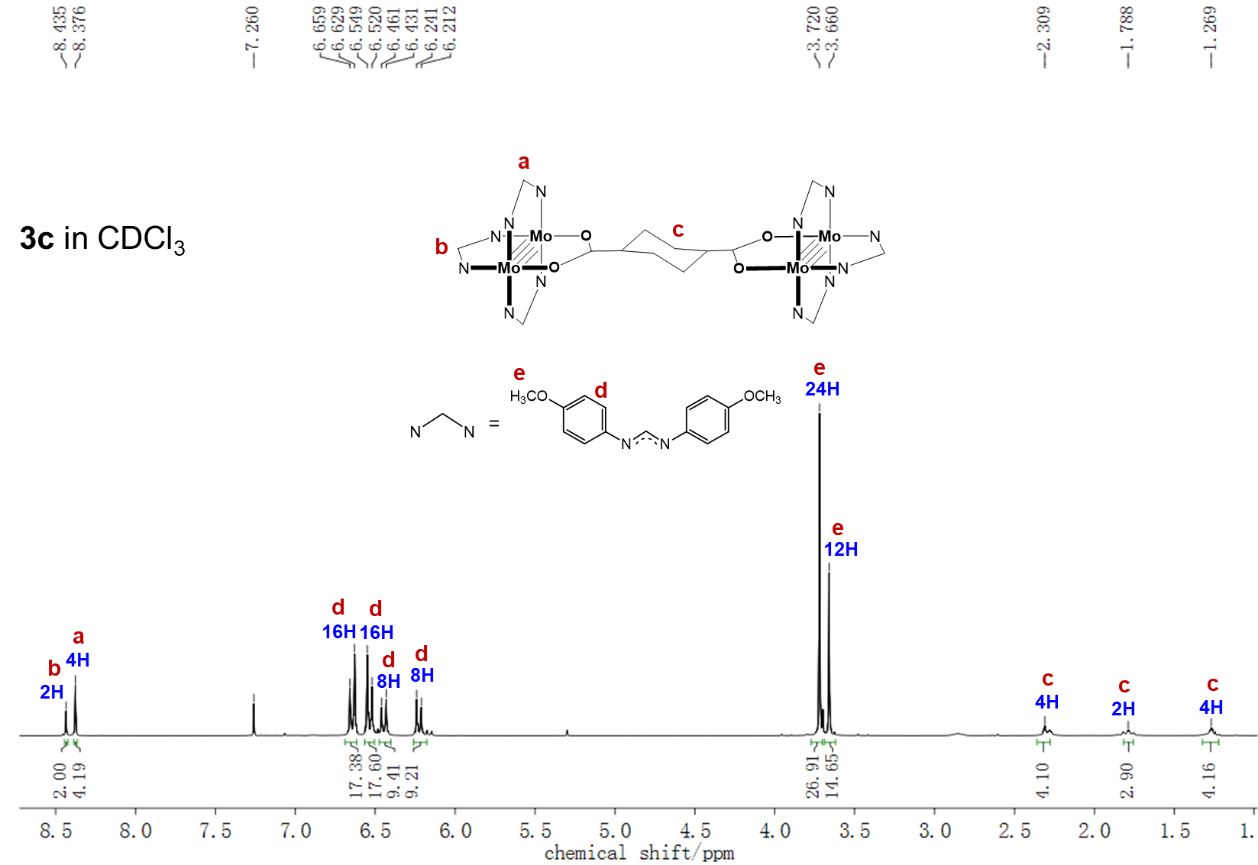


**Supplementary Figure 29.** ^1^H NMR spectrum of complex **3c** in CDCl_3_.

**3d**: Yield = 0.151g, 80 . ^1^H NMR *δ*(ppm in CDCl_3_): 8.499 (s, 2H, −NC*H*N−), 8.457 (s, 4H, −NC*H*N−), 6.855 (d, 16H, aromatic C−*H*), 6.661 (d, 8H, aromatic C−*H*), 6.477 (d, 16H, aromatic C−*H*), 6.199 (d, 8H, aromatic C−*H*), 2.224 (s, 24H, −C*H*_3_), 2.155 (s, 12H, −C*H*_3_), 2.802 (m, 4H, −C*H*_2_), 2.013 (m, 2H, −C*H*), 1.741 (m, 4H, −C*H*_2_).


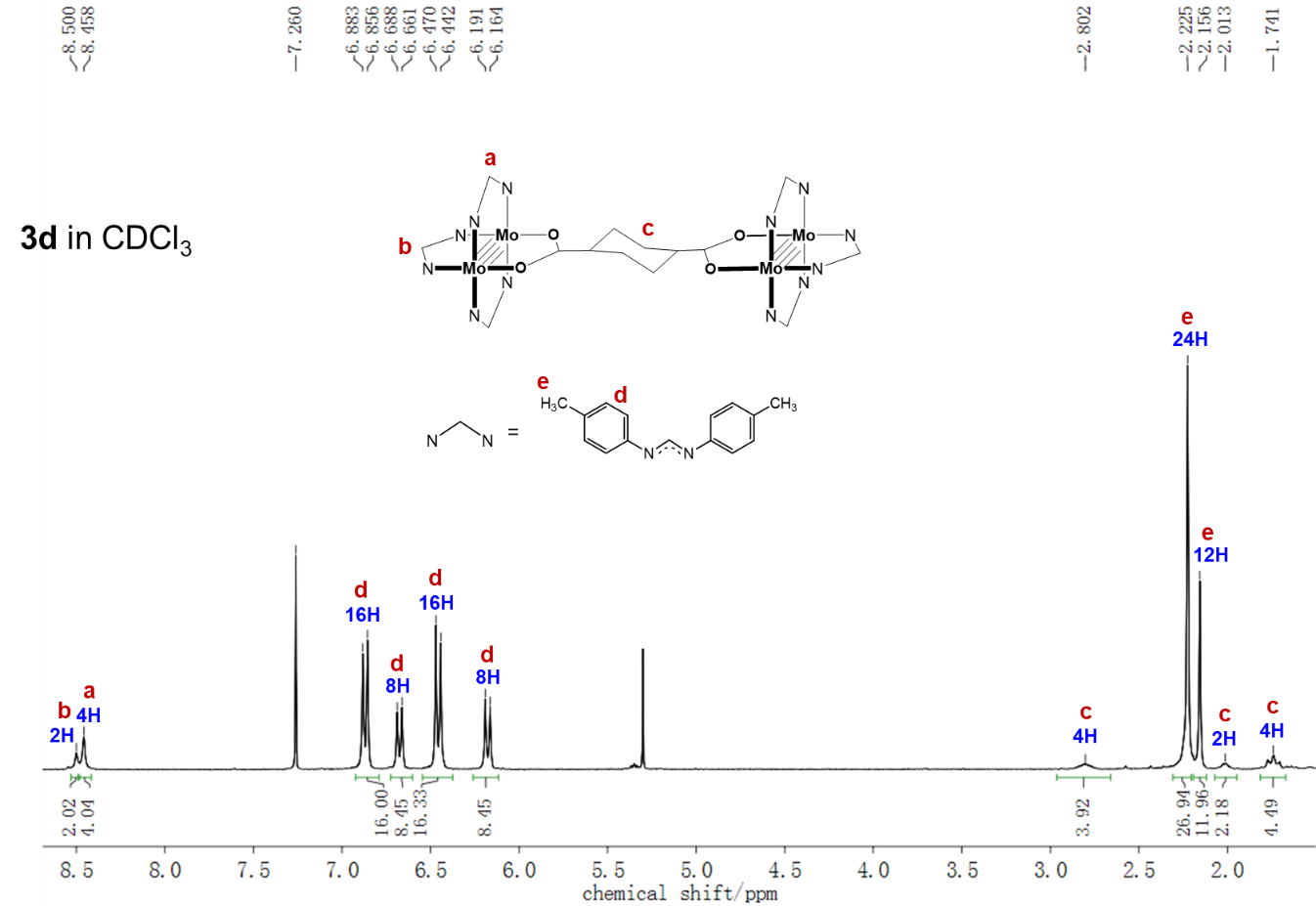


**Supplementary Figure 30.** ^1^H NMR spectrum of complex **3d** in CDCl_3_.

**Supplementary Table 1.** Crystallographic Data for complexes **1a**−**d**.

|  | **1a·7**CH_2_Cl_2_ | **1b·7**CH_2_Cl_2_ | **1c** | **1d·**CH_2_Cl_2_ |
| --- | --- | --- | --- | --- |
| Formula | C_113_H_144_Cl_14_Mo_4_N_26_  O_6_ | C_125_H_154_Cl_14_Mo_4_N_14_O_6_ | C_47_H_47_Mo_2_N_7_O_9_ | C_94_H_96_Cl_2_Mo_4_N_14_O_6_ |
| FW | 2842.61 | 2828.69 | 1045.80 | 2326.29 |
| space group | *P*ī | *P*ī | *P*2_1_/c | *P*ī |
| *a*(Å) | 11.48164(19) | 11.8919(5) | 14.4072(2) | 10.33963(8) |
| *b*(Å) | 13.6052(3) | 16.0571(6) | 17.3922(2) | 20.69259(17) |
| *c*(Å) | 22.2940(5) | 20.3413(6) | 18.3138(2) | 25.96649(18) |
| *α*(deg) | 77.3980(19) | 106.565(3) | 90.00 | 102.4787(6) |
| *β*(deg) | 75.2341(17) | 94.713(3) | 95.2520(10) | 98.6056(6) |
| *γ*(deg) | 73.5215(16) | 109.829(4) | 90.00 | 103.0597(7) |
| *V*(Å^3^) | 3189.01(12) | 3432.6(3) | 4569.68(10) | 5167.78(8) |
| *Z* | 1 | 1 | 4 | 2 |
| *T* (K) | 150 | 150 | 173 | 150 |
| *d*_calcd_(g/cm^3^) | 1.480 | 1.368 | 1.520 | 1.4949 |
| *μ*(mm^-1^) | 6.345 | 5.866 | 5.025 | 6.734 |
| *R*_1_*^a^* | 0.0614 | 0.1273 | 0.0358 | 0.0489 |
| *wR*_2_*^b^* | 0.1743 | 0.3162 | 0.0935 | 0.1390 |
| *^a^R*_1_ =**Σ**||*F*_o_| −|*F*_c_||/**Σ**|*F*_o_|. *^b^wR*_2_ = [**Σ**[*w*(*F*_o_^2^−*F*_c_^2^)^2^]/**Σ**[*w*(*F*_o_^2^)^2^]]^1/2^ | | | | | |

**
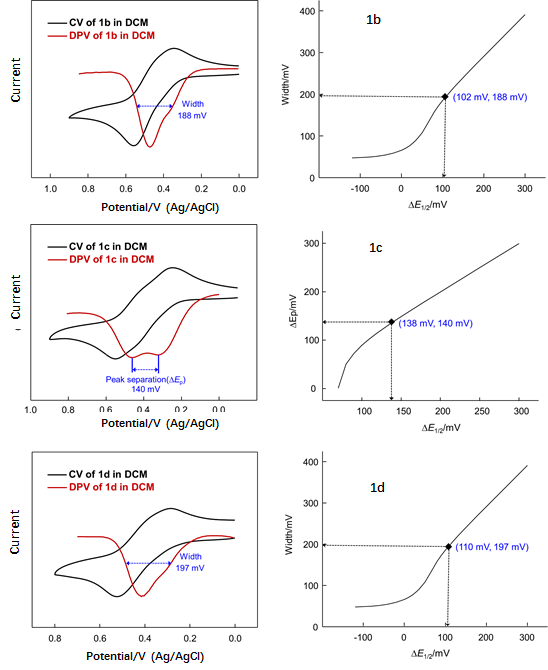
**

**Supplementary Figure 31.** Diﬀerential pulse voltammograms (DPVs, red) and cyclic voltammograms (CVs, black) for complexes **1b**−**d** in 0.1M *^n^*Bu_4_NPF_6_ /DCM solutions. The Δ*E*_1/2_ value are estimated from the working curve (width versus Δ*E*_1/2_) based on the Richard−Taube method.


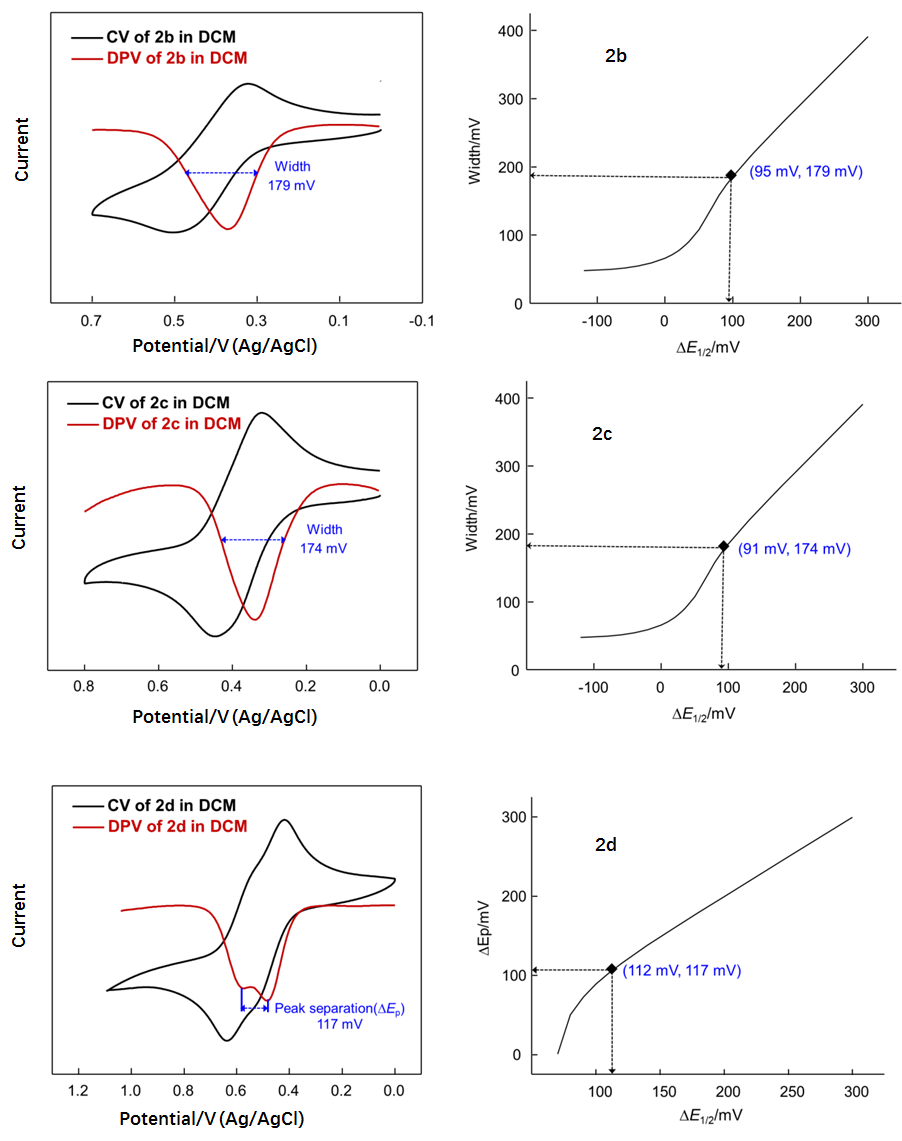


**Supplementary Figure 32.** Differential pulse voltammograms (DPVs, red) and cyclic voltammograms (CVs, black) for complexes **2b**−**d** in 0.1M *^n^*Bu_4_NPF_6_ / DCM solutions. The value of Δ*E*_1/2_ is estimated from the working curve (width versus Δ*E*_1/2_) based on the Richard−Taube method.


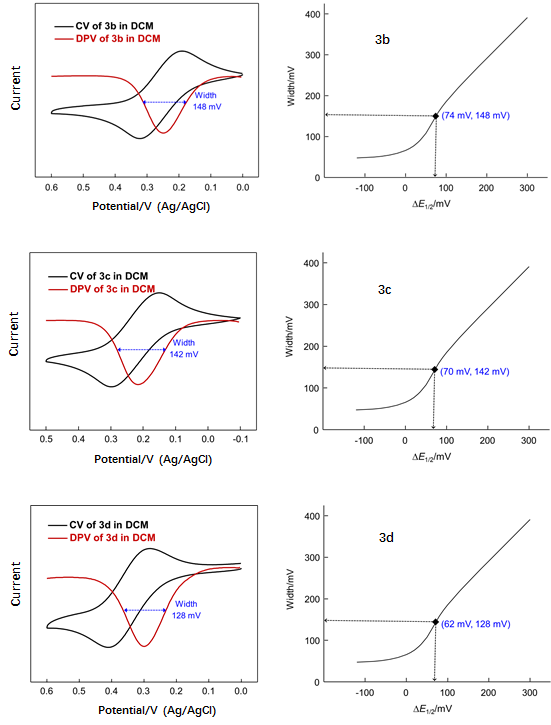


**Supplementary Figure 33.** Differential pulse voltammograms (DPVs, red) and cyclic voltammograms (CVs, black) for complex **3b**−**c** in 0.1M *^n^*Bu_4_NPF_6_ / DCM solutions. The value of Δ*E*_1/2_ is estimated from the working curve (width versus Δ*E*_1/2_) based on the Richard−Taube method.


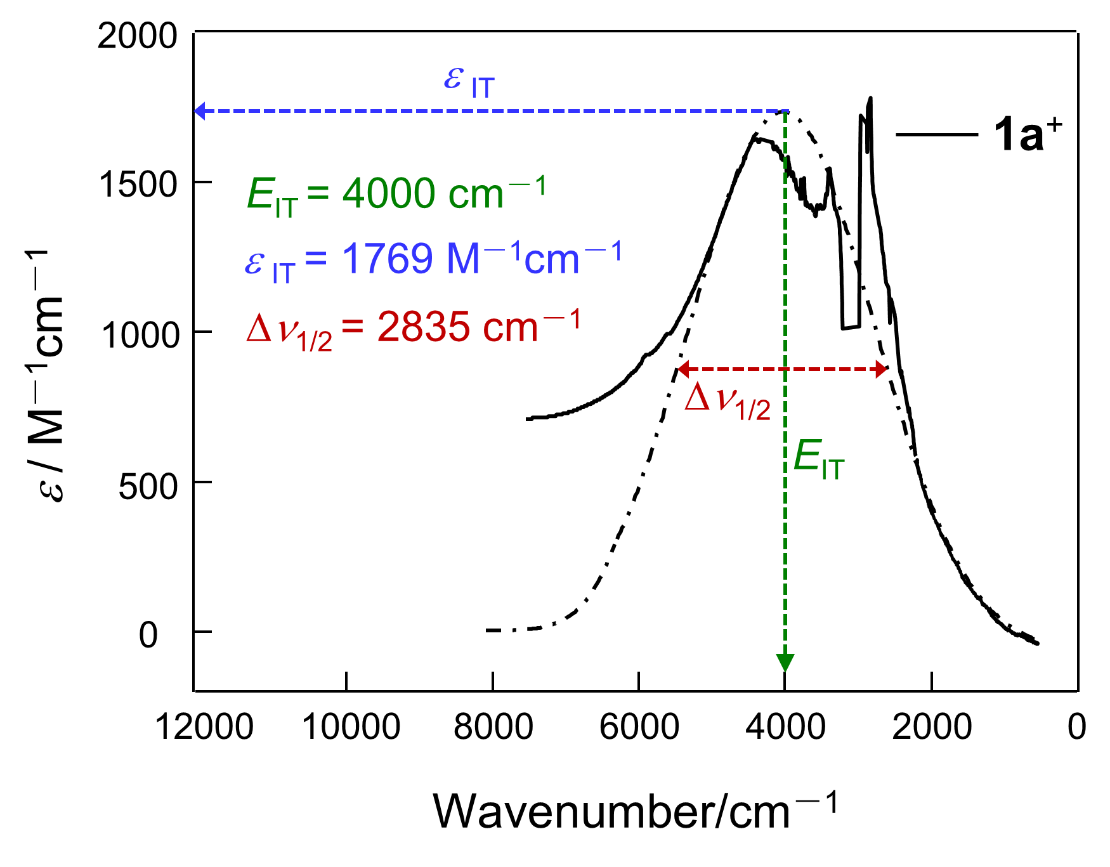


**Supplementary Figure 34.** Extraction of IVCT band parameters (*E*_IT_, *Ɛ*_IT_ and Δ*v*_1/2_) for the mixed-valence complex **1a**^+^ by Gaussian-shape simulation of the absorption band for calculation of *H*_ab_ parameter from Mulliken-Hush expression.


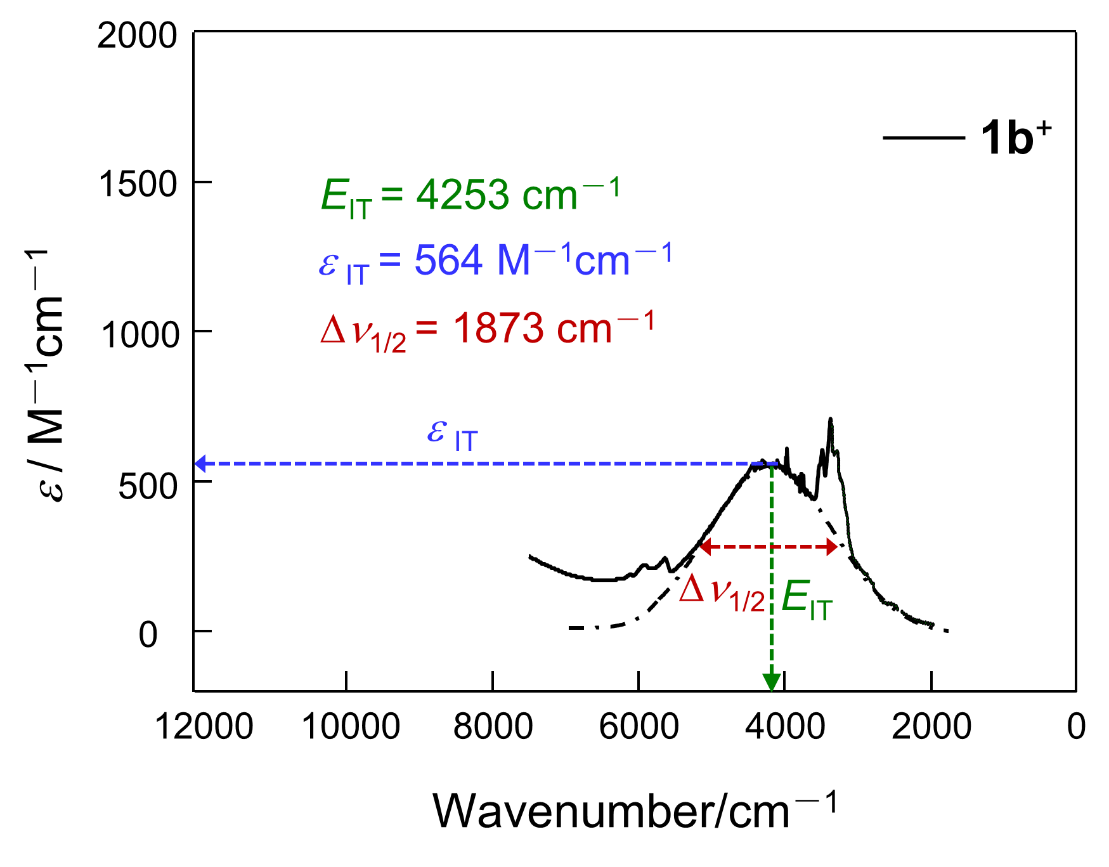


**Supplementary Figure 35.** Extraction of IVCT band parameters (*E*_IT_, *Ɛ*_IT_ and Δ*v*_1/2_) for the mixed-valence complex **1b**^+^ by Gaussian-shape simulation of the absorption band for calculation of *H*_ab_ parameter from Mulliken-Hush expression.


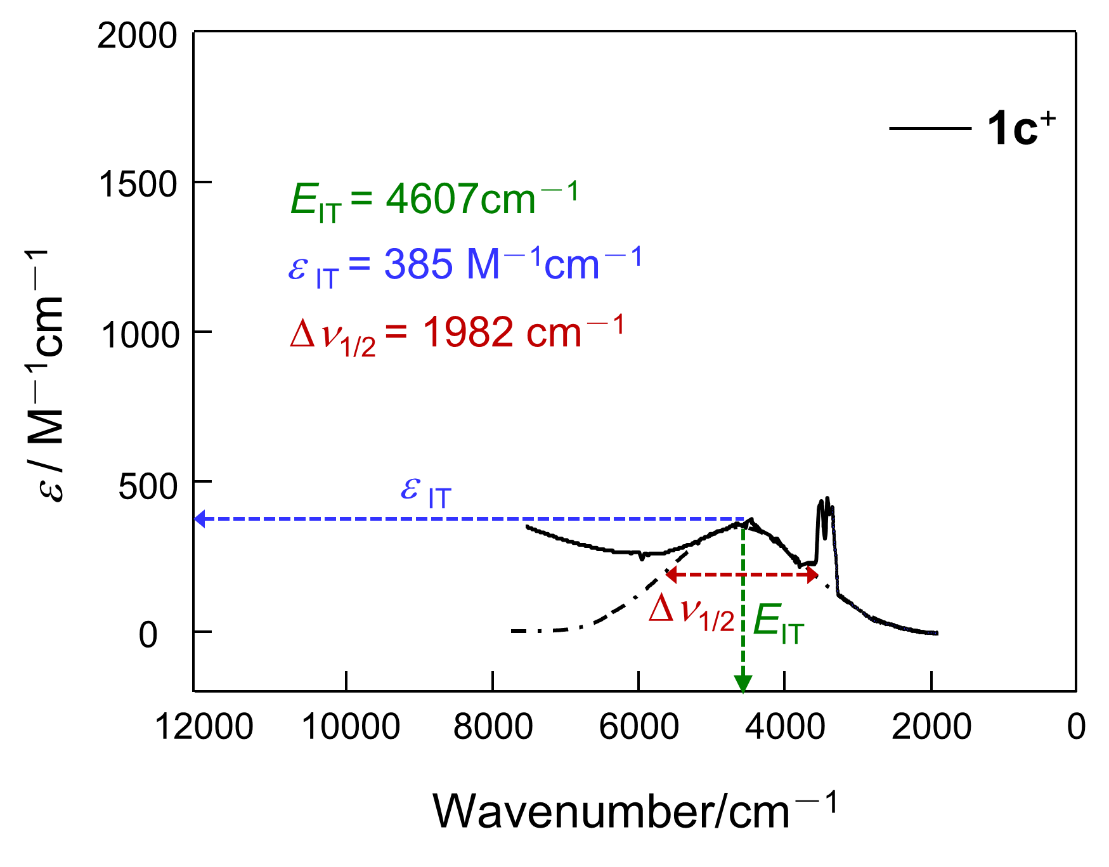


**Supplementary Figure 36.** Extraction of IVCT band parameters (*E*_IT_, *Ɛ*_IT_ and Δ*v*_1/2_) for the mixed-valence complex **1c**^+^ by Gaussian-shape simulation of the absorption band for calculation of *H*_ab_ parameter from Mulliken-Hush expression.


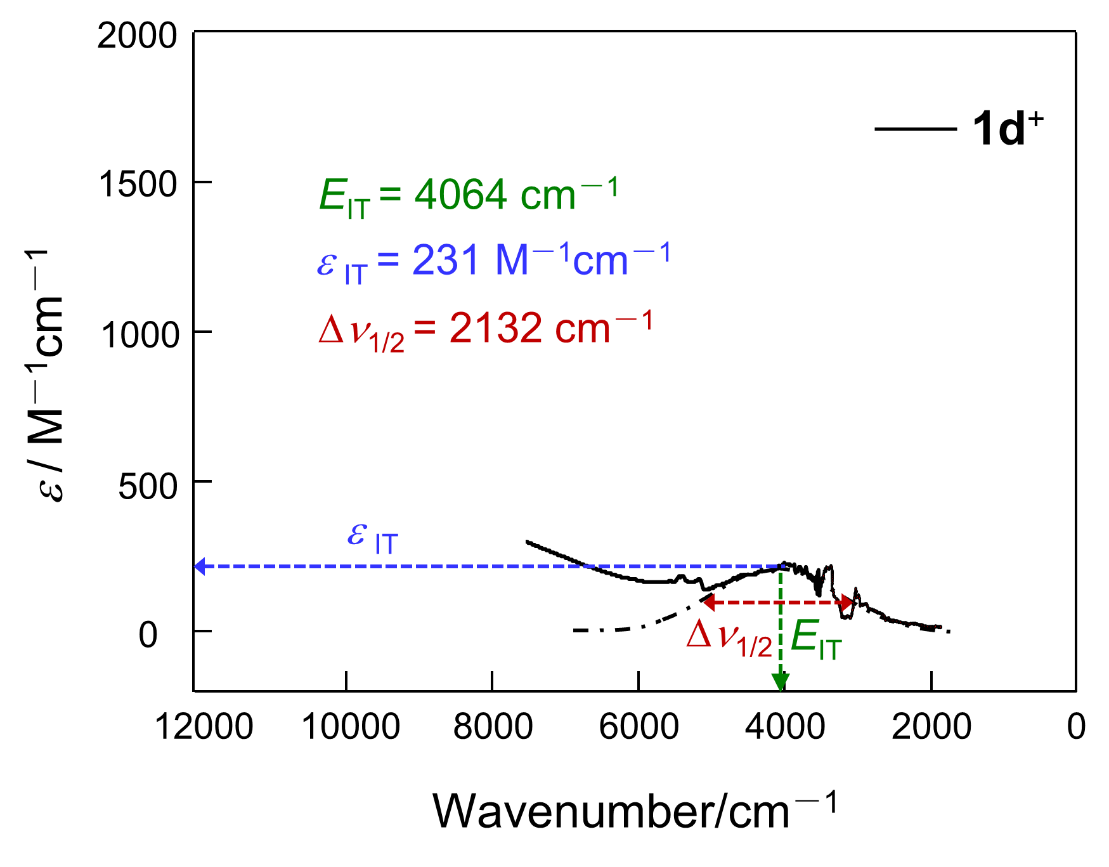


**Supplementary Figure 37.** Extraction of IVCT band parameters (*E*_IT_, *Ɛ*_IT_ and Δ*v*_1/2_) for the mixed-valence complex **1d**^+^ by Gaussian-shape simulation of the absorption band for calculation of *H*_ab_ parameter from Mulliken-Hush expression.


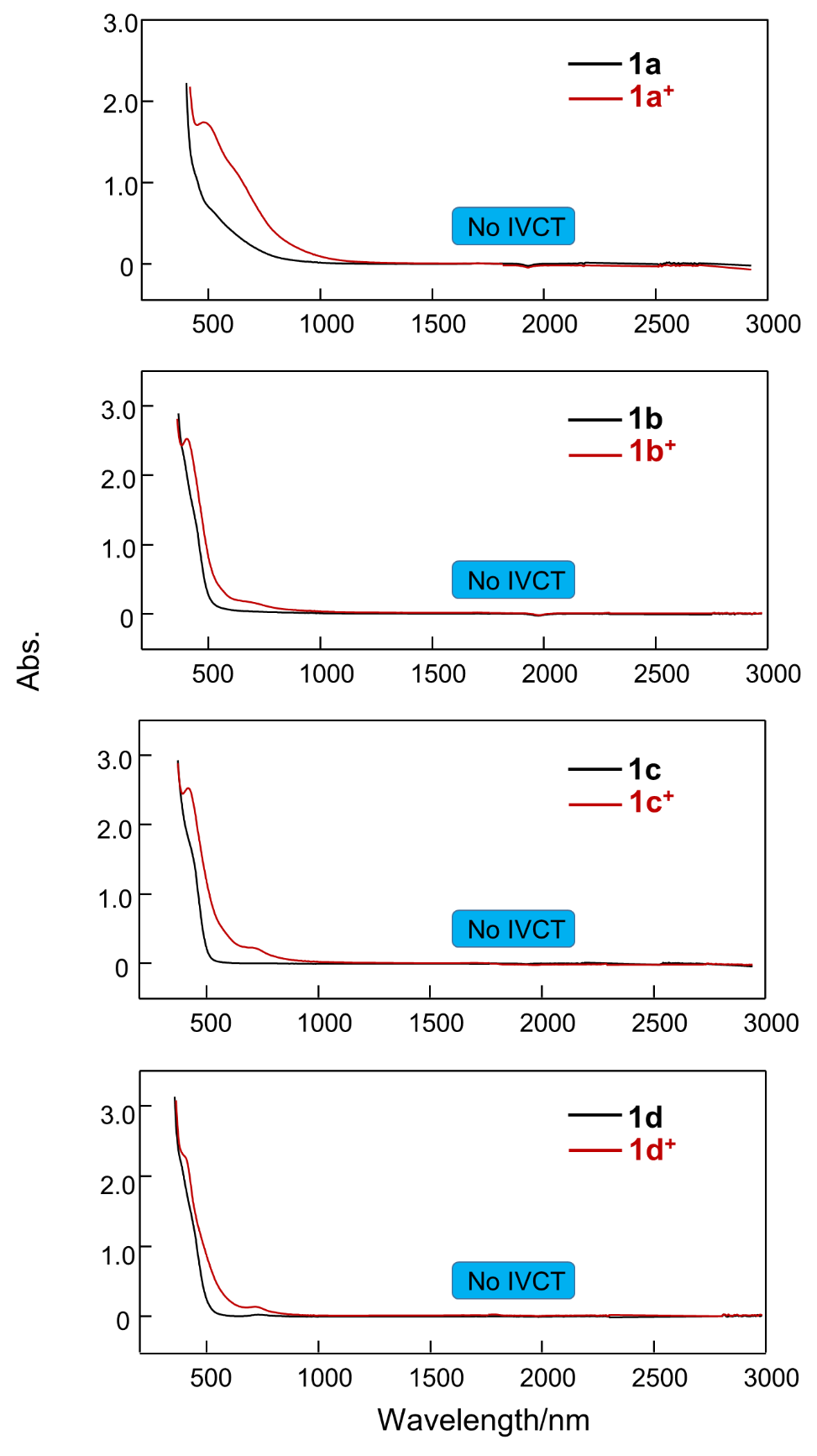


**Supplementary Figure 38.** UV-vis-near-mid-IR spectra of the radical complexes (**1a**−**d**)^+^ (red) and their corresponding neutral precursor**s** (**1a**−**d**, black) in DMF solutions. Note that in DMF, an IVCT transition is not observed for all these complexes.


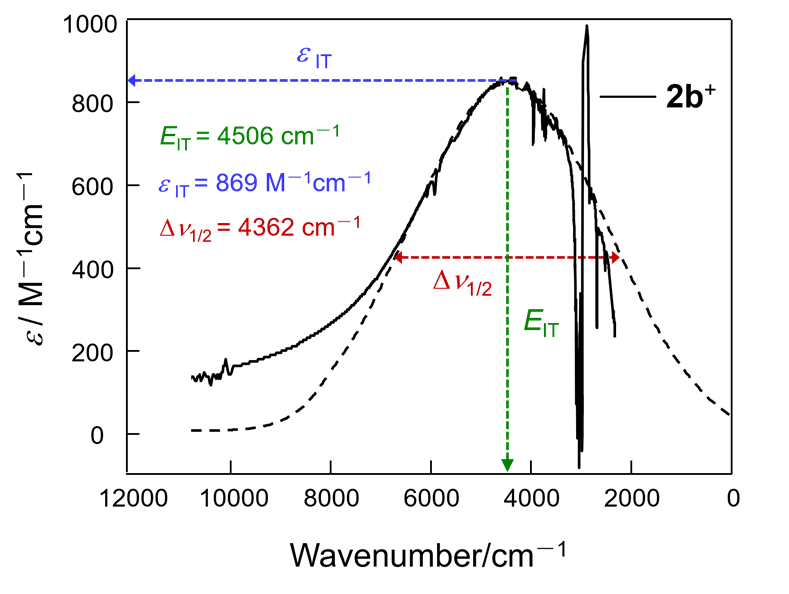


**Supplementary Figure 39.** Extraction of IVCT band parameters (*E*_IT_, *Ɛ*_IT_ and Δ*v*_1/2_) for the mixed-valence complex **2b**^+^ by Gaussian-shape simulation of the absorption band for calculation of *H*_ab_ parameter from Mulliken-Hush expression.


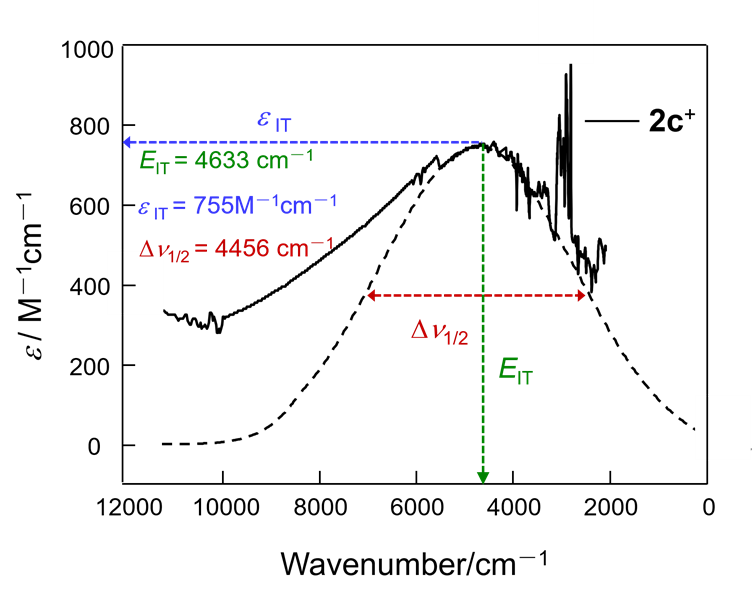


**Supplementary Figure 40.** Extraction of IVCT band parameters (*E*_IT_, *Ɛ*_IT_ and Δ*v*_1/2_) for the mixed-valence complex **2c**^+^ by Gaussian-shape simulation of the absorption band for calculation of *H*_ab_ parameter from Mulliken-Hush expression.


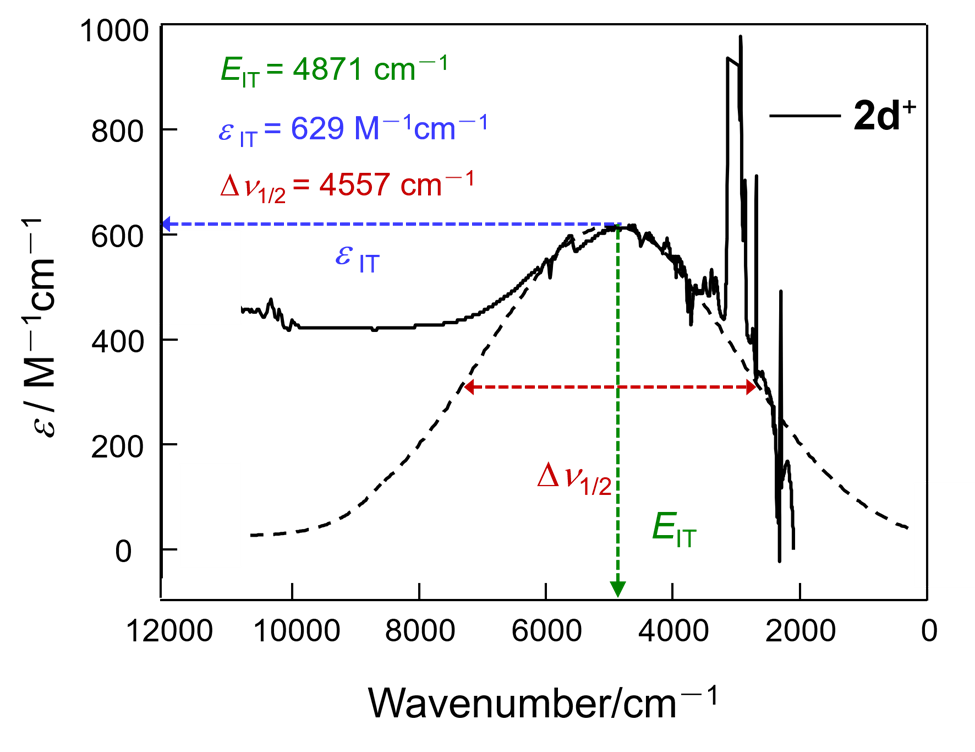


**Supplementary Figure 41.** Extraction of IVCT band parameters (*E*_IT_, *Ɛ*_IT_ and Δ*v*_1/2_) for the mixed-valence complex **2d**^+^ by Gaussian-shape simulation of the absorption band for calculation of *H*_ab_ parameter from Mulliken-Hush expression.


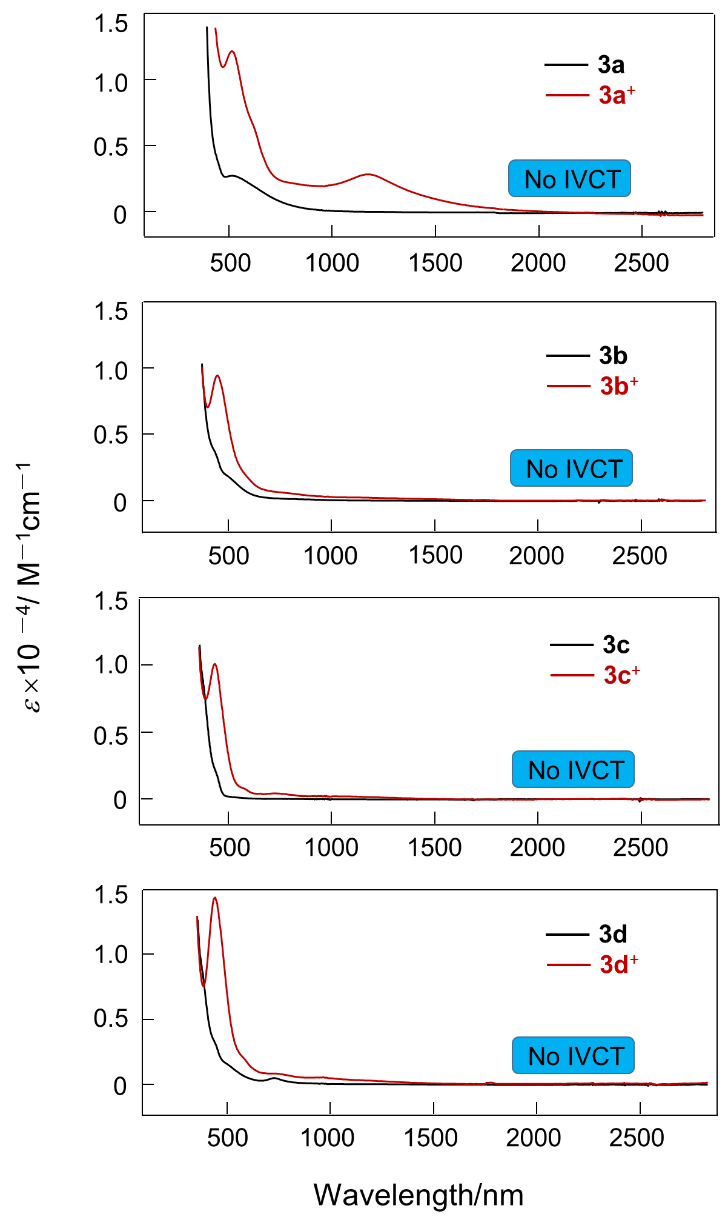


**Supplementary Figure 42.** Comparison of the vis-near-mid-IR spectra for the mixed-valence complexes (3**a**−**d**)^+^ (red) and their corresponding neutral precursors (**3a**−**d**, black) in DCM solutions. The spectra show no evidence of an IVCT absorption band, due to the weak electronic coupling between [Mo_2_] units.


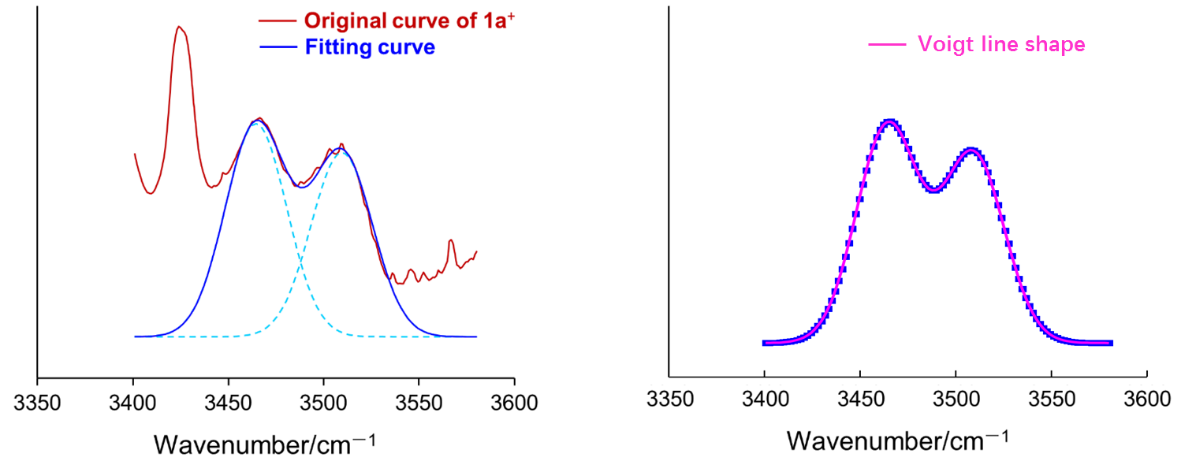


**Supplementary Figure 43.** Spectral fitting (left) and Voigt simulation (right) for the N−H vibrational bands of the MV complex **1a**^+^.


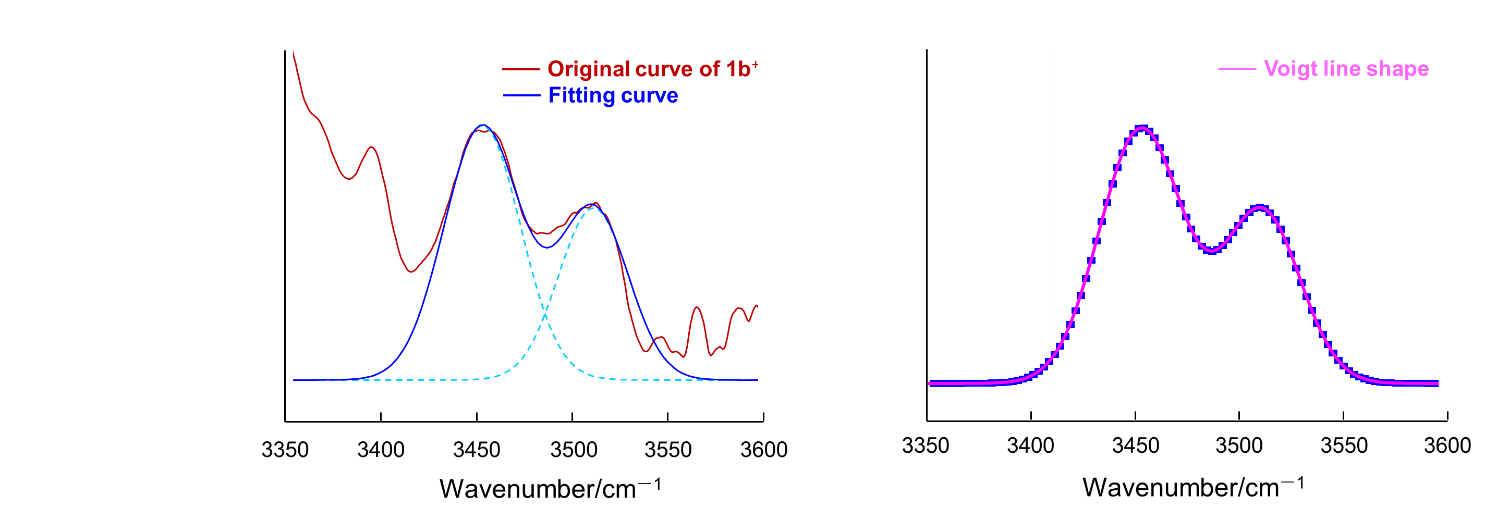


**Supplementary Figure 44.** Spectral fitting (left) and Voigt simulation (right) for the N−H vibrational bands of the MV complex **1b**^+^.


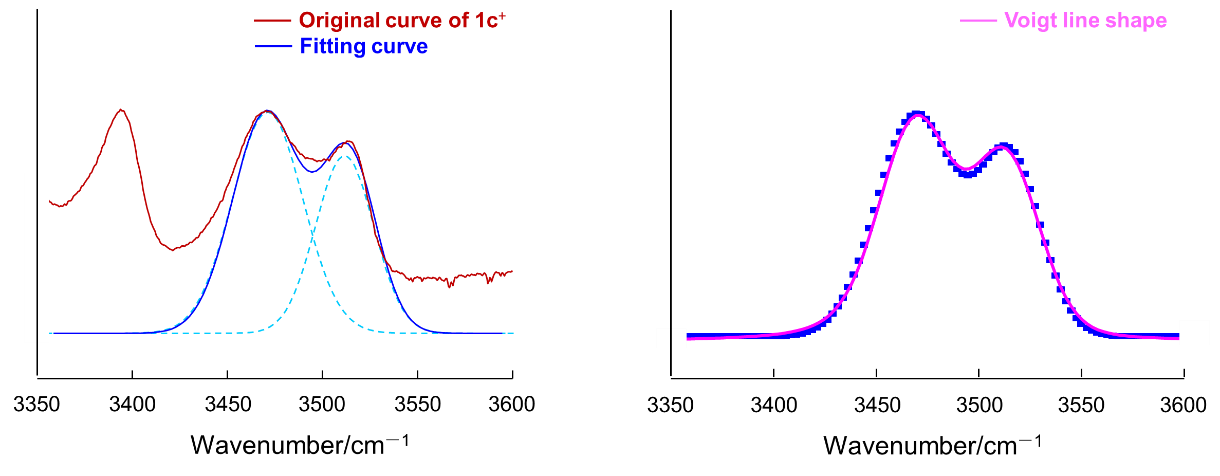
**Supplementary Figure 45.** Spectral fitting (left) and Voigt simulation (right) for the N−H vibrational bands of the MV complex **1c**^+^.


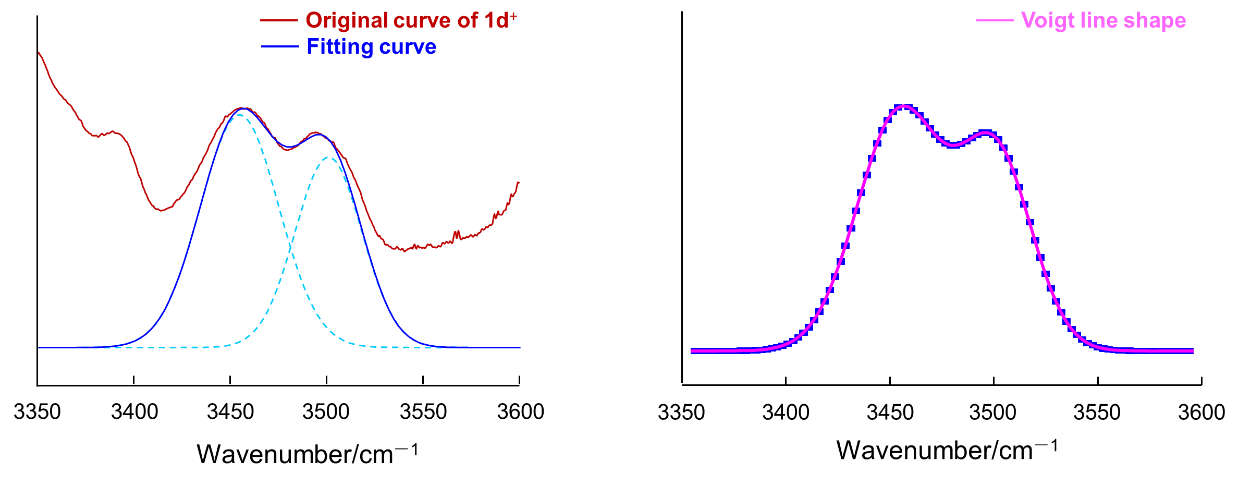


**Supplementary Figure 46.** Spectral fitting (left) and Voigt simulation (right) for the N−H vibrational bands of the MV complex **1d**^+^.

**Supplementary Table 2.** Fitting parameters for the N−H vibrational bands of complexes (**1a**−**d**)^+^ using Voigt function obtained from *Origin* software

| Complex | center frequency  (cm^−1^) | | population  (%) | | | Gaussian width  (cm^−1^) | | Lorentzian width (cm^−1^) | |  |
| --- | --- | --- | --- | --- | --- | --- | --- | --- | --- | --- |
|  | Peak 1 | Peak 2 | Peak 1 | | Peak 2 | Peak 1 | Peak 2 | Peak 1 | Peak 2 | |
| **1a^+^** | 3465 | 3510 | 56 | 44 | | 38 | 37 | 2.2×10^−6^ | 6.2×10^−13^ | |
| **1b^+^** | 3453 | 3511 | 62 | 38 | | 47 | 43 | 1.0×10^−11^ | 2.1×10^−19^ | |
| **1c^+^** | 3470 | 3514 | 64 | 36 | | 46 | 34 | 1.3×10^−5^ | 6.0×10^−14^ | |
| **1d^+^** | 3455 | 3501 | 59 | 41 | | 48 | 41 | 8.5×10^−8^ | 6.9×10^−14^ | |

**SUPPLEMENTARY REFERENCES**

1. Zhu, G. Y., Meng, M., Tan, Y. N., Xiao, X. & Liu, C. Y. *Inorg. Chem*. **55**, 6315−6322(2016). [↑](#endnote-ref-1)
2. Xiao, X., Meng, M., Lei, H. & Liu, C. Y. *J. Phys. Chem. C* **118**, 8308−8315(2014). [↑](#endnote-ref-2)
3. Shriner, R. L. & Neumann, F. W. *Chem. Rev*. **35**, 351−425(1944). [↑](#endnote-ref-3)
4. Cotton, F. A., Liu, C. Y., Murillo, C.A., Villagrán, D. & X. Wang, *J. Am. Chem. Soc*. **125**, 13564−13575(2003). [↑](#endnote-ref-4)
